# Integrated multispectral remote sensing and field investigations for delineating ophiolitic complexes of Wadi Ghadir southeastern desert Egypt

**DOI:** 10.1038/s41598-025-32272-8

**Published:** 2026-01-06

**Authors:** Moamen M. Badr, Ahmed M. El Mezayen, Ashraf El Azab, Mahmoud H. Elyaseer

**Affiliations:** 1 Geology Department, Faculty of Science, Al-Azhar University, PO Box 11884, Nasr City, Cairo Egypt; 2 Nuclear Materials Authority, P.O. Box 530, El Maadi, Cairo, Egypt

**Keywords:** Landsat-9, Sentinel-2, Ophiolite, Wadi ghadir, Multispectral remote sensing, Environmental sciences, Natural hazards, Solid Earth sciences

## Abstract

This study addresses the long-standing challenge of accurately distinguishing ophiolitic rock units in the structurally complex terrains South Eastern Desert of Egypt’s. By integrating multispectral satellite data from Sentinel 2 and Landsat 9 (OLI 2) with substantial field investigation and petrographic analyses, this research proposes an enhanced methodological framework for precise lithological mapping of the Wadi Ghadir ophiolitic complex. Advanced enhanced digital image processing methods for False Color Composites (FCC), Band Ratios (BR), Minimum Noise Fraction (MNF), Principal Component Analysis (PCA), and Maximum Likelihood (ML) classification were systematically applied and evaluated. A newly proposed band ratio combination of Landsat-9 (6/7, 6/5, 6/3) proved highly effective in differentiating serpentinite, talc-carbonate, metagabbro, sheeted dykes, and pillow lavas, while PCA and MNF transforms improved separation of granitic, metasedimentary, and volcanic units. The resulting 1:25,000 scale geological map demonstrates the innovation of integrating complementary satellite sensors with ground validation to overcome spectral confusion and mapping limitations typical of Neoproterozoic ophiolitic terranes. This integrated workflow enhances geological interpretation accuracy and provides a cost effective, reproducible approach for regional mineral exploration and mapping in similar environments.

## Introduction

 A well-defined ophiolite sequence is recognized in El Fawkhir, El Ghadir, Abu Meriewa and Gerf areas within the Egypt Eastern Desert^1–5^. Sheeted dykes is extremely limited in the Central area of the Eastern Desert, with exposures restricted to Southern part of Eastern Desert^6^. In cases where ophiolitic sequences are well-preserved, they generally include a basal serpentinized ultramafic mantle unit, followed by layered and massive gabbros, a network of sheeted dykes, and capped by pillow basalts^7–11^. Ophiolites in the Egyptian Eastern Desert are found as tectonically deformed bodies or incorporated within ophiolitic mélanges^12^. Structural deformation has resulted in the absence of one or more lithologies in many Egyptian ophiolites^13^. Ophiolites are either in the form of nappes or mélanges, with the latter identified by serpentinite and metabasalt assemblages. The lower sections are predominantly made up of harzburgite, while lherzolite is rarely observed. Harzburgites commonly show evidence of deformation and have undergone alteration to form serpentinite and talc-carbonate assemblages^14–17^. An ophiolitic mélange of Wadi Ghadir region is comprising fragments of varying sizes embedded within a sheared matrix of pelitic material and serpentinite^7^.In Wadi Ghadir most ophiolitic units Mostly dismembered and disrupted, with complete or coherent sequences being relatively uncommon^10^. This succession comprises serpentinized peridotites, layered gabbros, massive gabbros with rosette structures, microgabbros, sheeted dykes, and pillow basalts^8,10,12^. The Wadi Ghadir ophiolite is interpreted to have developed within back arc basin, situated over a northeast of dipping subduction zone^2,7,10^.

Recent progress in remote sensing methods and digital image processing tools has significantly improved the ability to distinguish lithological units in complex and highly deformed terrains. Several state-of-the-art (SoA) examinations have demonstrated the efficiency of multispectral datasets. Landsat, Sentinel-2, and ASTER in delineating ophiolitic and metavolcanic units through techniques like band ratios, PCA, and MNF transformations. These approaches have significantly improved lithological mapping accuracy and reduced field-based uncertainties in ophiolitic settings. The effectiveness of SoA methodologies has been highlighted in recent works, for example^18^and^19^, which confirm the potential of integrating multi-sensor data for enhanced geological interpretation.

This study builds upon recent research on Neoproterozoic ophiolitic sequences and regional tectonics, integrating field, petrographic, and remote sensing analyses to further advance the understanding of mantle-crust interactions in the Southern Eastern Desert^20,21^.

This research integrates newly acquired Landsat-9 (OLI-2) and Sentinel 2 imagery data with field observation in detail and petrographic analyses to enhance 1:25,000-scale lithological mapping of the Wadi Ghadir ophiolitic complex. It proposes new band ratio combinations for distinguishing ophiolitic rocks and demonstrates a novel, reproducible, and cost-effective workflow that overcomes spectral confusion between mafic and ultramafic units, thereby optimizing field efforts and improving geological mapping accuracy in complex terrains.

Lithological discrimination in highly heterogeneous and structurally intricate ophiolitic terrains cannot be reliably achieved using a single image-processing technique. Each method emphasizes distinct spectral or spatial characteristics; therefore, a multi-algorithm framework was essential for the present study. False Color Composites (FCC) enhance visual lithological contrasts, facilitating manual delineation of fundamental rock contacts. Band Ratios (BR) suppress topographic effects and accentuate absorption features associated with ferromagnesian minerals that typify ophiolitic assemblages. Principal Component Analysis (PCA) minimizes band redundancy and isolates spectral variance attributable to lithological differences, while the Minimum Noise Fraction (MNF) transformation distinguishes coherent geological signals from atmospheric and sensor-related noise. Finally, supervised Maximum Likelihood Classification (MLC) provides statistically robust, pixel-based lithological discrimination grounded in field-verified training samples.

To reduce spectral ambiguity and enhance classification accuracy, it is not necessary to rely on a single technique. Instead, combining multiple methods can be particularly effective, especially when dealing with Neoproterozoic ophiolitic complexes.

## Geologic setting

Egyptian Nubian Shield (ENS) encompasses an area of approximately 100,000 square kilometers and is prominently exposed in certain areas in the Southwestern Desert (specifically Oweinat area), Southern part of Sinai, and the Egyptian Eastern Desert alongside Red Sea on the Egyptian side^22^.

**Fig. 1:**
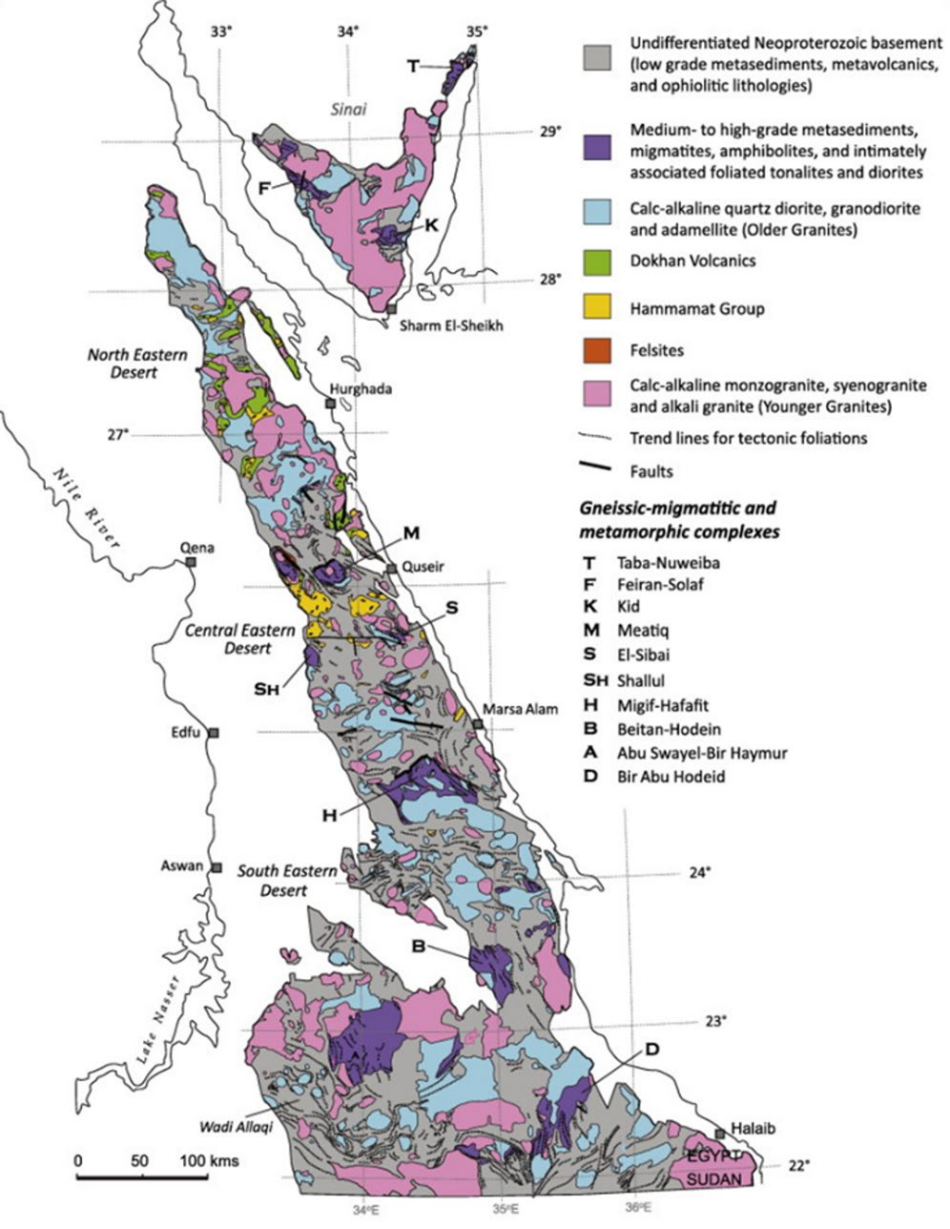
Image shows the lithological of the Egyptian Nubian Shield (ENS)^53^ (By ArcGIS v.10.5. https://www.esri.com/en-us/arcgis/products/arcgis-desktop/overview).

The ENS comprises four major lithological components, as depicted in (Fig. 1): mainly juvenile arc assemblages, supracrustal sequences, ophiolitic complexes, and gneissic domains that host core complexes with granitoid intrusions. The tectonic amalgamation of these units resulted from thrusting linked to accretionary processes and left lateral strike-slip shearing, primarily with Najd Fault System and other northwest-trending shear zones, especially within the central region of the Egyptian Eastern Desert^23^. Sheared granitoid gneisses in Egypt’s Eastern Desert commonly appear in domal antiformal areas, surrounded by low grade supracrustal units like the Meatiq dome, Hafafit, El-Shalul, and the area along Wadi Beitan. Egypt was historically regarded as exposures of a pre Neoproterozoic basement complex. Alternatively, some researchers suggest that these gneisses are of juvenile origin, formed within continental arc or intra-oceanic environments, either in the Mozambique Ocean or along its western margin. They propose that these rocks developed along one or more magmatic arcs earlier to the convergence of West and East Gondwana around (630) million years ago. These interpretations are based on a range of studies and analyses conducted by various researchers^23–49^.

Stern and Hedge^50^ classified the Egyptian Eastern Desert of into three distinct regions. First, the North Eastern Desert (NED); then, the Centre Eastern Desert (CED); and finally, the South Eastern Desert (SED). These regions provide unique insights into the prolonged and intense Neoproterozoic period of igneous activity and deformity that has characterized the historical geology of the area. The study area within the Southeastern Desert (SED) is characterized by major structural features, most prominently the Allaqi-Heiani Suture (AHS) and the Hamisana Shear Zone (HSZ). The AHS forms part of the extensive Allaqi-Heiani-Oneib-Sol Hamed-Yanbu Suture (AHOSHYS) and trends approximately east-west for over 600 km. This suture separates the Hijaz–Gebeit terrane to the south from the Midyan–South-Eastern Desert terrane to the north.

**Fig. 2:**
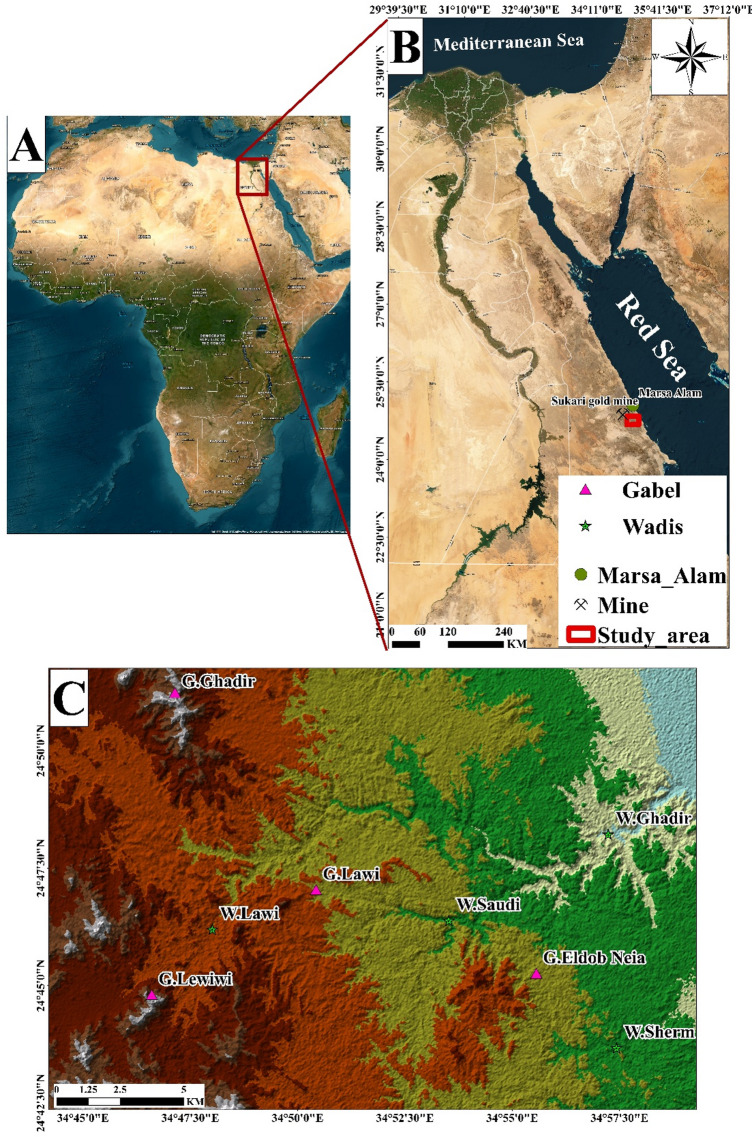
(**A**) Map showing Egypt’s location within the African. (**B**) Map illustrating the position of the study area within Egypt. (**C**) ALOS PALSAR DEM highlighting the Gabel Lawi area in the South Eastern Desert of Egypt. (By ArcGIS v.10.5. https://www.esri.com/en-us/arcgis/products/arcgis-desktop/overview).

Wadi Ghadir, situated in the Southern part of Sukari gold mine within Southeastern Desert. The geographical confines of the study area are demarcated by latitudinal coordinates ranging from 24°51′41″ to 24°49′57″ N and longitudinal coordinates spanning from 34°44′10″ to 34°59′19″ E. The study area covers about 457 Km^2^. Wadi Ghadir area, characterized by several wadis and mountains (Wadi Ghadir, Wadi Sherm, Wadi Lawi, Gabel Ghadir, Gabel El Dob Neia, Gabel Lawi, and Gabel Leweiwi) (Fig. 2), was selected for its well-preserved Neoproterozoic ophiolites and proximity to key structural features like Hamisana Shear Zone and Allaqi-Heiani Suture, providing an ideal site to study tectonic evolution, mantle-crust interactions, and mineral exploration potential. Most ophiolites of Egypt are significantly deformed and fragmented; however, some relatively intact Neoproterozoic ophiolite sections are preserved, notably in the Wadi Ghadir area^12,51,52^. The ophiolitic sequences in the area consist of serpentinized peridotites, both layered and isotropic gabbros, sheeted dyke complexes, and mafic massive and pillow lavas, sometimes overlain by pelagic sedimentary deposits. The Wadi Ghadir ophiolite, situated near the junction of Wadi Beda and Wadi El Saudi, was initially mapped and described by^12^. This consists of serpentinites, metagabbros, metabasalts, sheeted dyke complexes, and associated pelagic sedimentary rocks. Some pillow structures are preserved as isolated, brecciated fragments, with individual pillows displaying characteristic features such as a convex shaped upper surface, a holocrystalline interior, and vesicle-enriched margins^12^.

## Materials and methods

This study combined remote sensing analysis with field validation and a comprehensive review of academic resources including peer-reviewed journal articles, conference papers, textbooks, geological maps, reports, and authoritative institutional websites to support the research framework.

**Fig. 3:**
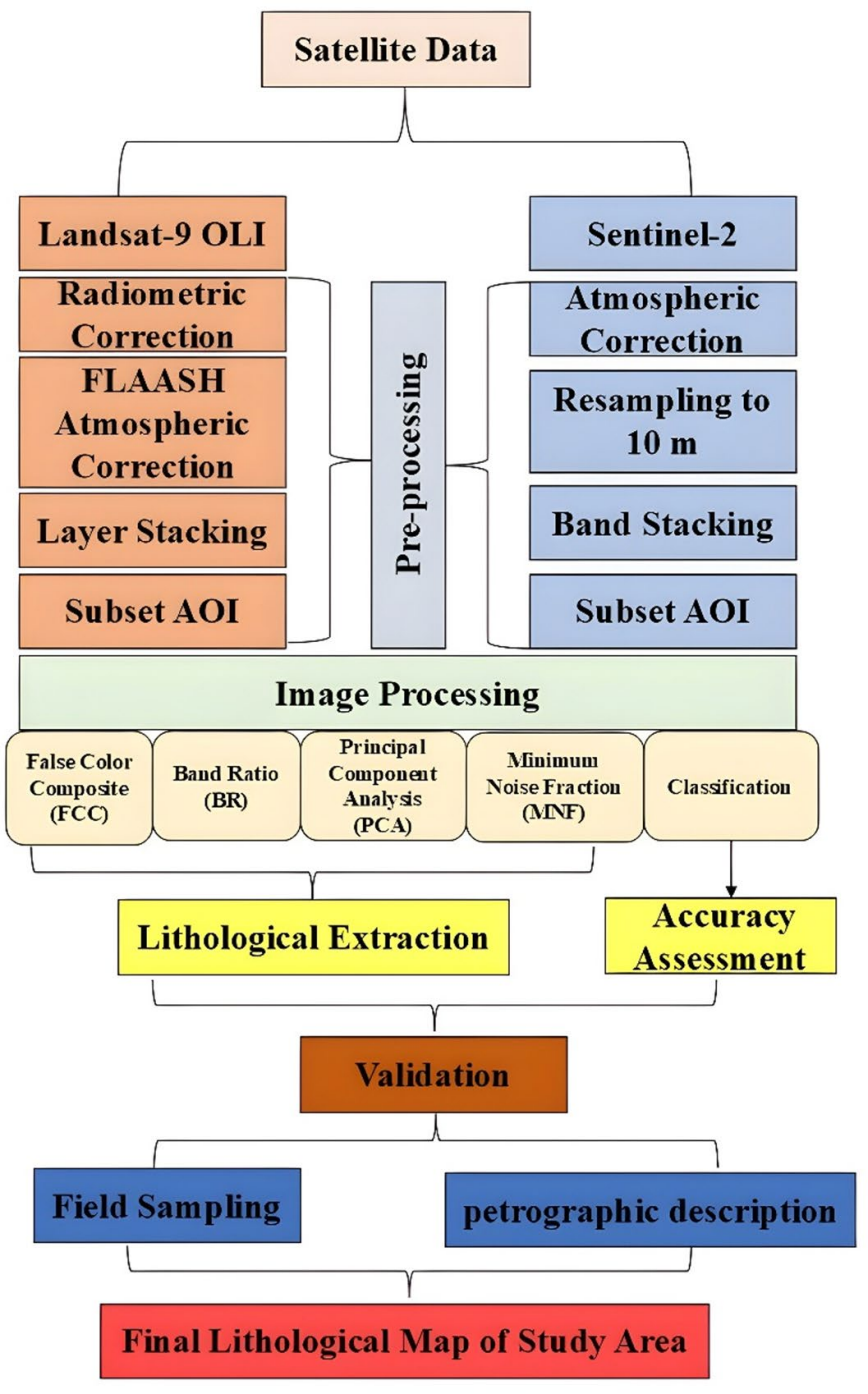
Methodology flowchart for this study.

As illustrated in (Fig. 3), the remote-control flow in this study consists of two main steps: preprocessing and processing, Sentinel-2 and Landsat- 9 systems used to disrupt ophiolitic complexes and their principal changes and lithologies. In the pre-processing stage, radiometric and atmospheric corrections have been applied to improve images accuracy before the extraction of the aimed information. Then advanced image processing techniques were employed to identify the geological features.

On September 27, 2021, Landsat-9 entered orbit. The satellite carried the Operational Land Imager-2 (OLI-2), while the Thermal Infrared Sensor-2 (TIRS-2) was produced by NASA Goddard Space Flight Center, Greenbelt, Maryland. Northrop Grumman was responsible for the development, construction, and integration of the spacecraft and its onboard instruments.

The Landsat-9 instruments are better versions of the Landsat 8 instruments, which are already producing data that is better in terms of geometry and radiometric than those on earlier Landsat satellite generations.

Equipped with TIRS-2 and OLI-2, Landsat-9 provides advanced Earth observation capabilities. The TIRS-2 instrument senses thermal infrared emissions from the planet’s surface, whereas OLI-2 captures reflectance data in the visible, near-infrared, and shortwave infrared ranges. The satellite’s improved radiometric performance enables it to resolve 16,384 unique brightness levels. https://www.usgs.gov/landsat-missions/landsat-9.

Sentinel 2 mission is a high- resolution multispectral imaging project designed to broad Earth observing as part of European Space Agency’s Copernicus Program. It consists of twin satellites in the same orbit, 180 degrees apart, which enables a regular return cycle of about five days at the equator. (3) bands at 60 m, (6) bands at 20 m, and (4) bands with a 10-meter spatial precision are among the 13 spectral bands that Sentinel-2’s advanced optical instrument capacity can capture data in. The mission’s 290-kilometer orbital sweep breadth ensures wide surface coverage with each pass https://sentinels.copernicus.eu/copernicus/sentinel-2.

This study utilized a set of advanced processing methods, including PCA, FCC, MNF, BR, and ML classification, for enhance the interpretation and analysis of remote sensing data^54^. These techniques were conducted on Landsat-9 and Sentinel-2 imagery to enhance lithological differentiation and to complement field-based geological mapping efforts.

### Remote sensing analysis

#### Preprocessing methods

This study utilizes Landsat-9 (OLI-2) Collection 2 (Level 1) imagery, specifically covering Wadi Ghadir within row 43, path 173, projected in UTM Zone 36 within WGS-84 as the datum. The selected scene was acquired on October 5, 2023. Landsat-9 have 11 spectral bands; for this analysis, the VNIR bands and SWIR bands (bands 1–7), varying from (0.43 μm) to (2.29 μm) (Table 1), were employed. Additionally, Sentinel-2 A level 1 C imagery was incorporated to enhance the study region. The adopted Level 1 C of Sentinel-2 A imagery, comprising 12 spectral bands (Table 1), was acquired on November 3, 2023. To achieve optimal results, several software packages were utilized in this study, including ENVI 5.6, ArcGIS 10.8, ArcGIS Pro 3.3.2, and SNAP.

The first step is conversion to radiance; Landsat-9 is commonly represented as Digital Numbers (DNs), each DN corresponds to the electromagnetic radiation intensity (EMR). The second step is atmospheric correction which was carried out using the Fast Line of sight Atmospheric Analysis of Hypercubes (FLAASH) module of ENVI software to remove the effect of large molecules present in atmospheric gases such as ozone, water vapor, carbon dioxide and clouds. FLAASH incorporates several features including an adjustment for the adjacency effect, which addresses the blending of pixel values due to the diffusion of radiance reflected from nearby surfaces. It also includes the ability to estimate average scene visibility, reflecting the presence and density of atmospheric aerosols or haze^55^.The Level 1 C of Sentinel-2 image needs several steps to ensure radiometric and geometric corrections, ortho rectification, and generation of quality masks. The Sentinel Applications Platform (SNAP) software is free and useful for resampling grid computation, resampling of spectral bands and quality masks computation as preprocessing techniques.

**Table 1:** Spectral-Bands of Landsat-9-OLI-and-Sentinel-2 satellites.

Landsat 9 (OLI)			Sentinel-2		
Bands	Wavelength (micrometers)	Resolution (meters)	Bands	Wavelength (micrometers)	Resolution (meters)
Band-1 Coastal aerosol	0.43–0.45	30	Band-1 Coastal aerosol	0.443	60
Band − 2 Blue	0.45–0.51	30	Band − 2 Blue	0.490	10
Band-3 Green	0.53–0.59	30	Band-3 Green	0.560	10
Band − 4 Red	0.64–0.67	30	Band − 4 Red	0.665	10
Band-5 (NIR)	0.85–0.88	30	Band-5 (VNIR)	0.705	20
Band-6 (SWIR)	1.57–1.65	30	Band-6 (VNIR)	0.740	20
Band-7 (SWIR)	2.11–2.29	30	Band-7 (VNIR)	0.783	20
Band-8 Panchromatic	0.50–0.68	15	Band-8 (VNIR)	0.842	10
Band − 9 Cirrus	1.36–1.38	30	Band − 8a (SWIR)	0.865	20
Band-10 (TIRS)	10.6–11.19.6.19	100	Band-9 (SWIR)	0.945	60
Band-11 (TIRS)	11.50–12.51.50.51	100	Band-10 (SWIR)	1.375	60
			Band-11 (SWIR)	1.610	20
			Band-12 (SWIR)	2.190	20

#### Processing methods

##### Fales color composition (FCC)

The selection of optimal three-band color composite images can be accomplished using the Optimum Index Factor (OIF) technique. Spectral bands with lower mutual correlation are generally more effective for visualization within the RGB color space, as they enhance contrast and feature discrimination^56^.

The OIF method, which employs a statistical approach, also aids in selecting better color composites for visual examination of images. This method considers both the distinction within individual bands and the coefficients between different bands^57^. The formula used for this purpose evaluates the suitability of band combinations for creating optimal color composite images^58^.

##### Spectral signatures

Spectral signatures consist of the combination of electromagnetic radiation that is reflected, absorbed, transmitted, or emitted by objects at different wavelengths, which helps in uniquely identifying those objects. When you graph the amount of electromagnetic radiation, typically the intensity of reflected light or reflectance as a percentage, over various wavelengths, the resulting connected points create a curve known as the material’s spectral signature or spectral response curve. To effectively analyze remote sensing images, it is critical to begin with a fundamental grasp of spectral signatures, which refers to how different land features like water, rocks, soils, and plants interact with various wavelengths of electromagnetic radiation.

The spectral signature of an object relates to the incident of electromagnetic radiation and the part of the electromagnetic spectrum it interacts with. Instruments like purpose built spectrometers are used to measure the energy that is reflected from the object. Multispectral datasets such as Landsat, ASTER, and Sentinel-2 have long been employed in geological and environmental studies due to their wide coverage and accessibility. This fine spectral resolution allows for accurate identification of mineral types, compositions, and alteration patterns, providing a significant advantage in lithological discrimination and mineral exploration^59,60^.

##### Band ratio (BR)

Band ratio technique is utilized to minimize the effects of topographic variability and to reduce brightness contrasts associated with differences in grain size^61^. This method is widely recognized for its effectiveness in lithological mapping and has been employed across various regions, including the Arabian-Nubian Shield. The selection of bands for generating ratio images is determined by the specific spectral properties of the rock types being analyzed^62^. The application of ratioing techniques yields the most effective differentiation of rock groups when utilizing combinations of short-wavelength spectral bands (e.g., B3/B1, B4/B1, or B4/B2), long-wavelength band ratios (e.g., B5/B7), and ratios combining one band from the short-wavelength group with one from the long-wavelength group (e.g., B5/B4 or B5/B3)^63,64^.

##### Principal component analysis (PCA)

PCA addresses the redundancy that is found in multispectral images, the OLI, and Sentinel-2, so elegantly. This problem occurs due to different spectral bands looking somewhat identical, which creates a high correlation between them. PCA solves this problem by updating the data with correlated values to a new set of uncorrelated principal component (PC) axes.

According to^65^ and^66^, principal component (PC) bands are uncorrelated and often easier to interpret and analyze than the original spectral data. Moreover, the number of PC bands generated is equal to the number of spectral bands input. The first principal component (PC1) holds the utmost variance, which is found affected mostly by surface topography, and sun positioning due to bright observation on the surface, uncovering important structural information. Every next component (PC2, PC3…) holds less and less varying value compared to the previous one, and spectra differences between visible light and infrared are emphasized.

##### Minimum noise fraction (MNF)

MNF transform can also serve as an effective method for reducing noise in the data. Initially, the process begins with the application of a forward MNF transformation. Subsequently, the spectral bands that retain coherent image information are identified through the examination of both the transformed images and their associated eigenvalues. Following this, an inverse MNF transform is conducted using a selected spectral subset comprising only the informative bands. Alternatively, rather than excluding the noisy bands, they may be smoothed prior to applying the inverse transformation. The MNF rotation output can be subset using eigenvalues; therefore, with hyperspectral data, there is no need to produce a hundred of band (float) output cube when only a couple of dozens are required. The most important aim of the work is to expose the rock units using the MNF transform.

##### Classification

In this case, pixels with the same spectral behavior are assigned labels and grouped together^64,66^. In multispectral classification, pixels are assigned to specific classes based on their data values. Pixels belonging to a certain class are defined according to conditions and threshold values^67^. The two most common approaches are: supervised classification where the classes are defined in accordance with the provided training data, and unsupervised classification which defines classes without any training procedure, frequently used of conjunction in hybrid systems^68^.

##### Remote sensing results

**Fig. 4:**
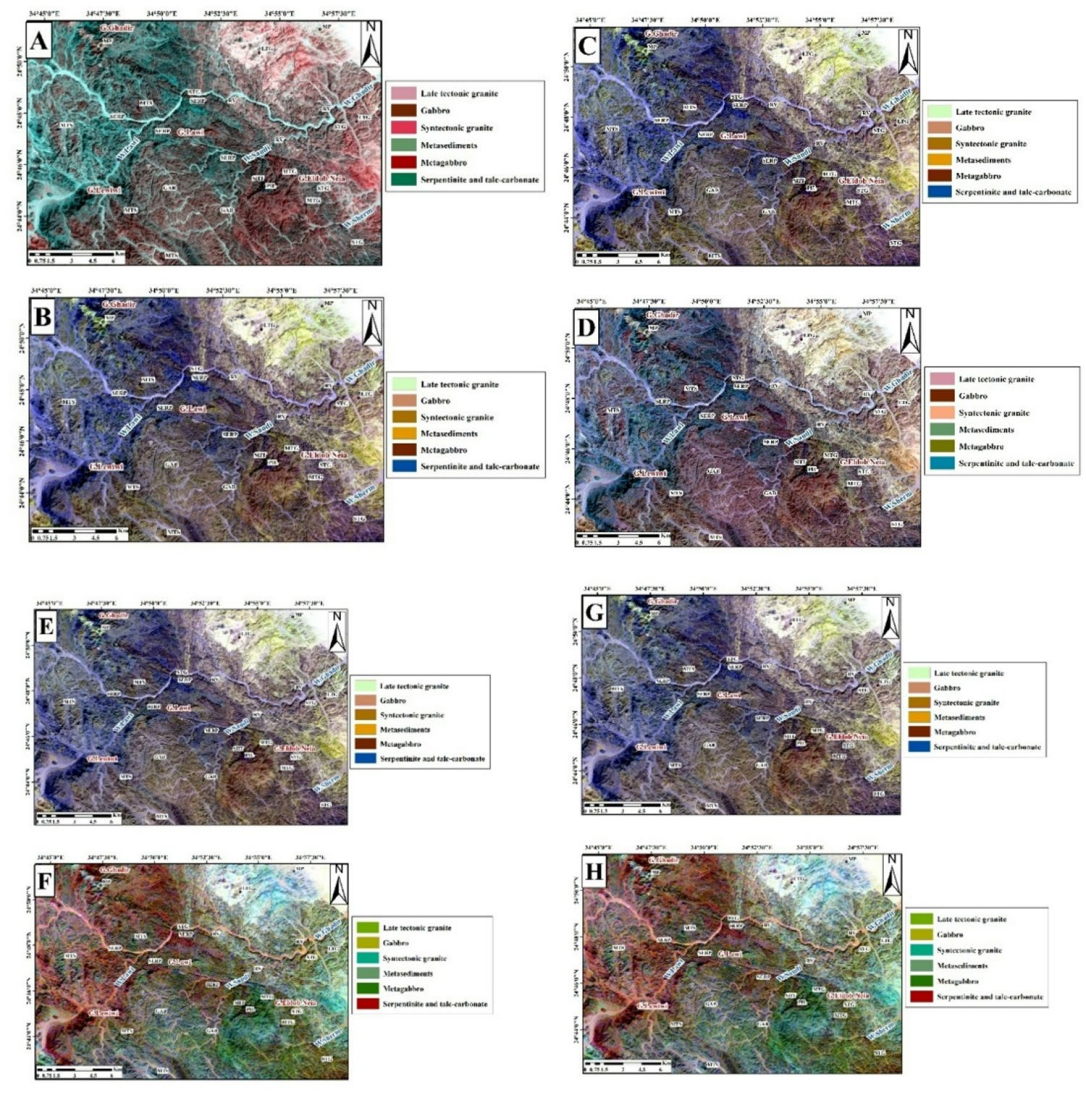
Landsat-9 FCC data were inspected in RGB respectively;7,2,1(**A**); 7,6,2 (**B**); and 7,6,1 (**C**); and7,5,3 (**D**). Sentinel-2 FCC as RGB images 2,11,10 (**E**); 2,13,11 (**F**); 3,11,10 (**G**); and 2,12,11(**H**). (By ENVI version 5.6. https://www.l3harrisgeospatial.com/Software-Technology/ENVI).

The OIF technique also was applied to Landsat-9, and Sentinel-2 images (Table 2). The most effective RGB combinations for Landsat-9 were 7,2,1 (Fig. 4A), 7,6,2 (Fig. 4B), and 7,6,1 (Fig. 4C), with the best visualization achieved using the 7,5,3 combination (Fig. 4D). Similarly, for Sentinel-2 data, the tested RGB combinations were 2,11,10 (Fig. 4E), 2,13,11 (Fig. 4F), and 3,11,10 (Fig. 4G), with the 2,12,11 combination (Fig. 4H) providing the clearest lithological contrast and overall best representation. These results indicate that the selected FCCs effectively highlight variations of rock units.

**Table 2:** Optimum index factor (OIF) ranking of (OLI), ASTER and Sentinel-2 data in the study area.

	Landsat-9		Sentinel-2	
Rank	Band triplet	OIF %	Band triplet	OIF %
1	7,2,1	70.77	2,11,10	85.59
2	7,6,2	70.35	2,13,11	83.01
3	7,6,1	70.06	3,11,10	82.29
4	7,5,3	69.25	2,11,1	82.21
5	7,3,2	69.15	2,12,11	80.89
6	7,3,1	68.95	3,11,1	80.57

**Table:** 

Symbols			
STG	Syntectonic granite	WD	Wadi deposits
FIM	Metavolcanics and their metapyroclastics	LTG	Late tectonic granite
MTS	Metasediments	GAB	Gabbro
PIL	Pillow lava		
SHT	Sheeted dykes		
MTG	Metagabbro		
SERP	Serpentinite and talc-carbonate		

**Fig. 5:**
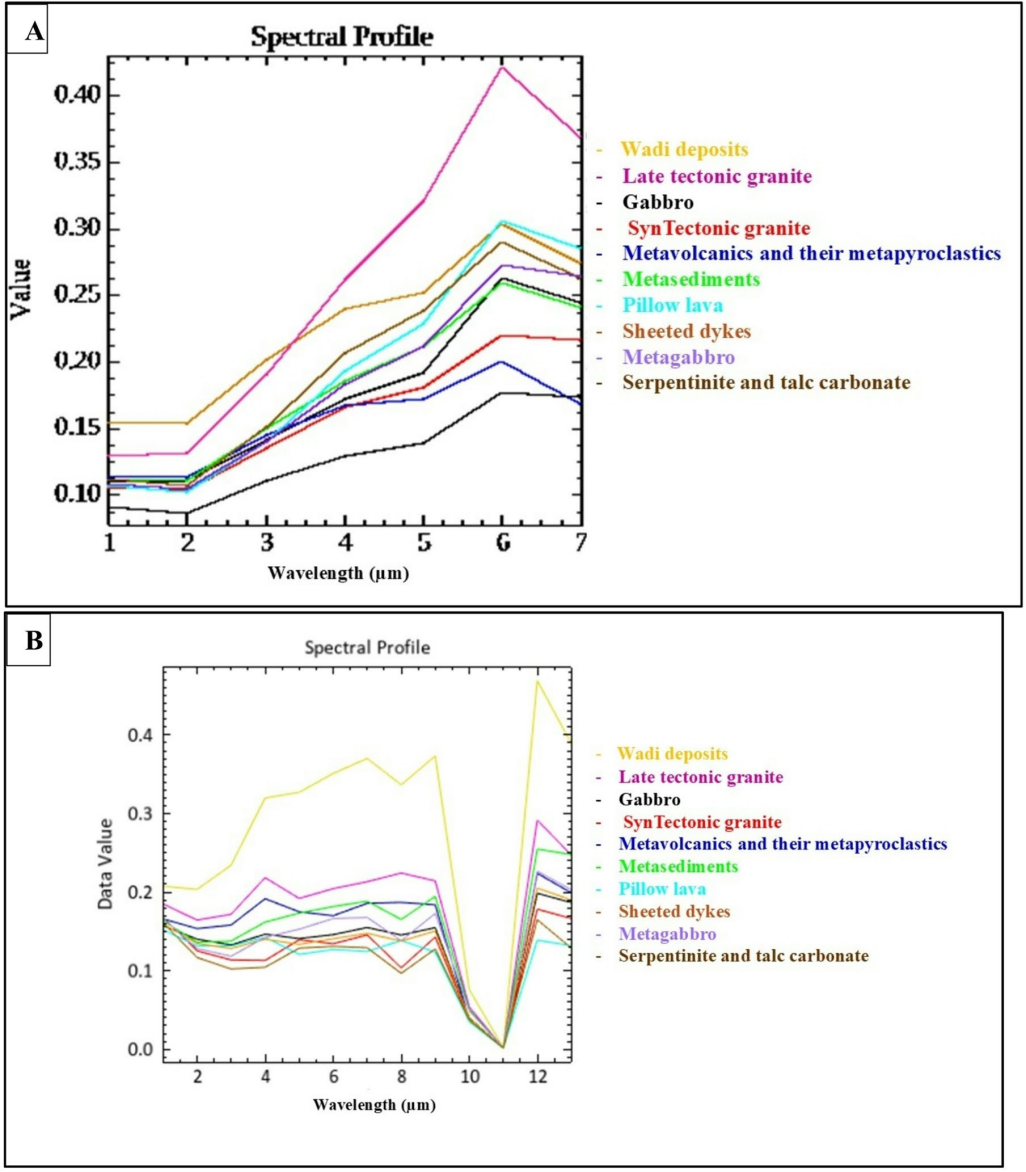
(**A**): Spectral Signatures of the rock units for Landsat-9. (**B**): Images showing Spectral Signatures of rock units for Sentinel-2.

Spectral signatures derived from Landsat-9 and Sentinel-2 data were conducted (Fig. 5A & B). The results revealed distinct spectral curves for the major lithological units, including serpentinite, metagabbro, and granitic rocks. Sentinel-2 data, with its higher spectral resolution in the VNIR and SWIR regions, provided more detailed and diagnostically sharper spectral profiles compared to Landsat-9, particularly for discriminating between different rock units.

**Fig. 6:**
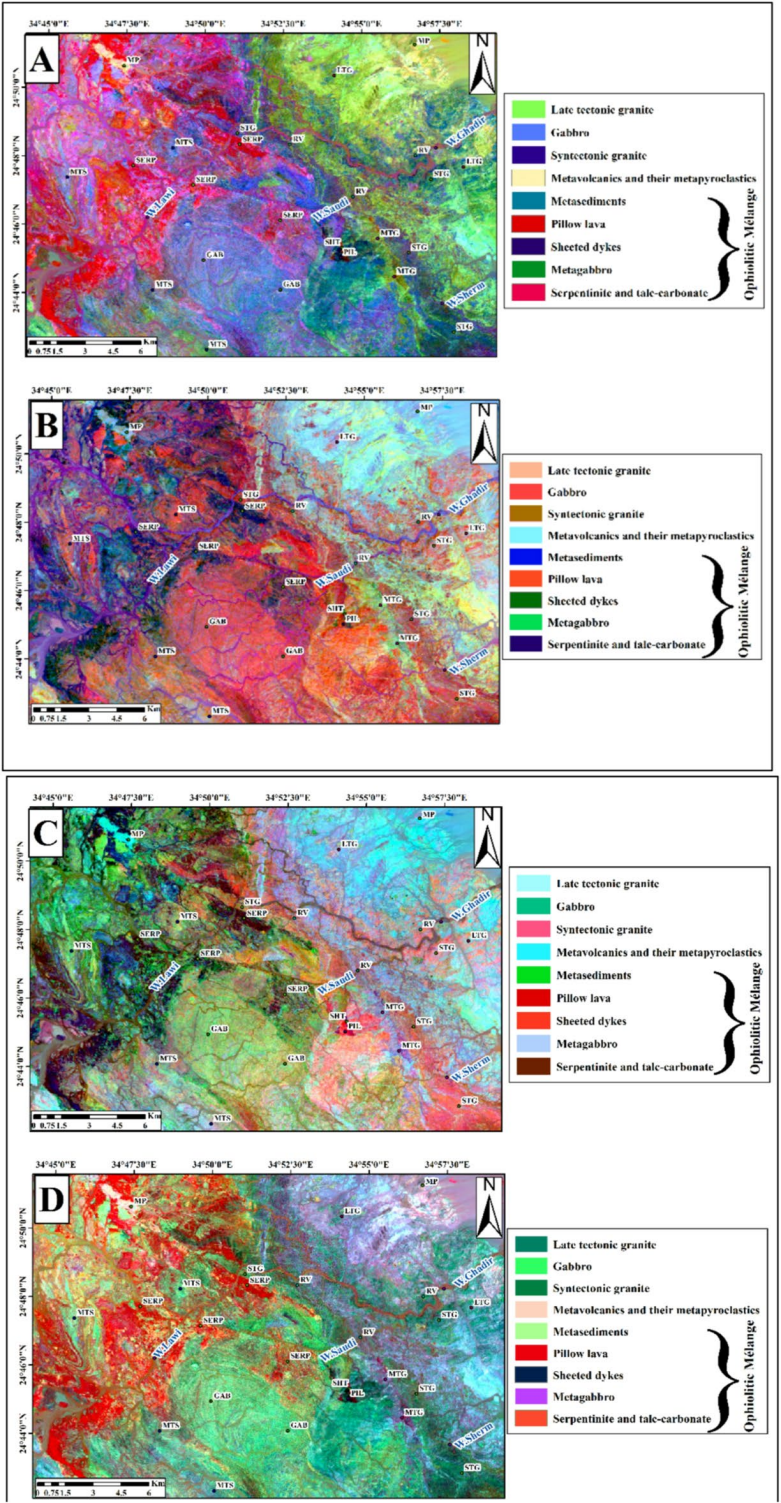
Landsat 9 OLI data were inspected that the RGB of ratios images. (**A**) Landsat-9 FCC band ratio (6/7, 6/2& 6/5*4/5) in RGB for the study area matching with^69^. (**B**) Landsat-9 FCC band ratio (7/5,5/3&3/1) in RGB for the study area (After^70^. (**C**) Landsat-9 FCC new band ratio differentiate between all rock units (7/6, 6/4 &5/4) in RGB for the study area. (**D**) Landsat-9 FCC new band ratio to separate sheeted dykes from pillow lava (6/7, 6/5& 6/3) in RGB for the study. Symbols as in Fig. 3. (By ENVI version 5.6. https://www.l3harrisgeospatial.com/Software-Technology/ENVI).

Band ratio techniques were applied to Landsat-9 data, following methodologies described by^69^, specifically, ratio combinations such as (6/7, 6/2, 6/5* 4/5) were utilized to enhance spectral contrasts and facilitate geological interpretations. This ratio shows serpentinite with hot pink color, metagabbro with green color, sheeted dykes with dark red color, pillow lava with dark blue color and metasediments with distant blue color, (Fig. 6A) that corresponding to (5/7, 5/1 and 5/4 * 3/4) used by^69,70^ suggested a ratio composite of (7/5, 5/3 & 3/1) in RGB respectively, from Landsat-8 bands to distinguish rock units at Wadi Hammamat area in the CED. Upon applying this ratio composite to the present study area, it was able to discriminate serpentinite with bluish green color, metagabbro with light green color, sheeted dykes with green color, pillow lava with orange color and metasediments with olive yellow color (Fig. 6B). In this study the authors suggested new ratios of Landsat-9 to differentiate between the different rock units, (7/6, 6/4 &5/4) where serpentinite shows dark brown color, metagabbro with pearl violet color, sheeted dykes with purple violet color, pillow lava with pink color and metasediments with green color (Fig. 6C). To separate sheeted dykes from pillow lava in this area the authors used this ratio (6/7, 6/5& 6/3) (Fig. 6D), where sheeted dykes show purple red color and pillow lava have dark green color.

**Fig. 7:**
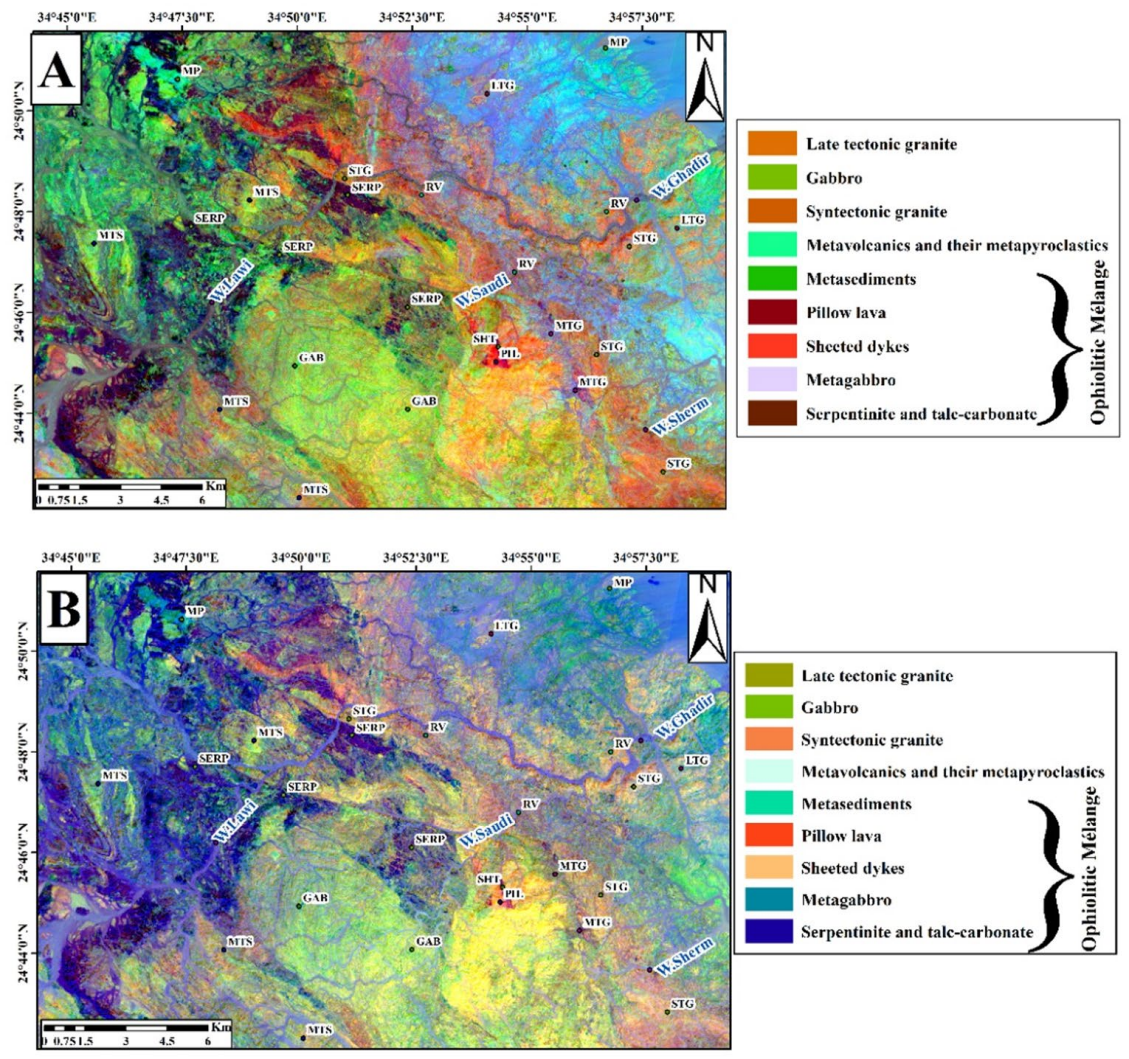
Sentinel-2 band ratio composites proposed in this study: (**A**) (13/12, 12/7& 4/3) in RGB, (**B**) (12/13, 13/6 &2/1) in RGB. Symbols are as in Fig. 3. (By ENVI version 5.6. https://www.l3harrisgeospatial.com/Software-Technology/ENVI).

Authors suggested different ratios to distinguish between different rock units using Sentinel-2 bands; the ratio composite of RGB (13/12, 12/7& 4/3) provided the best results in separating the following rocks, serpentinite with brown color, metagabbro with pearl violet color, sheeted dykes with pink color, pillow lava with orange color and metasediments with green color, (Fig. 7.A). Moreover, using this ratio (12/13, 13/6 &2/1) enabled separating serpentinites from metasediments, where serpentinite shows dark blue color, and metasediments exhibit green color (Fig. 7.B).

The focal point for applying PCA of Landsat-9 and Sentinel − 2 were delineating the surface-exposed rock units in the study area. The PCA analysis showed that these components contain the highest values of eigenvectors (Tables 3 and 4).

**Fig. 8:**
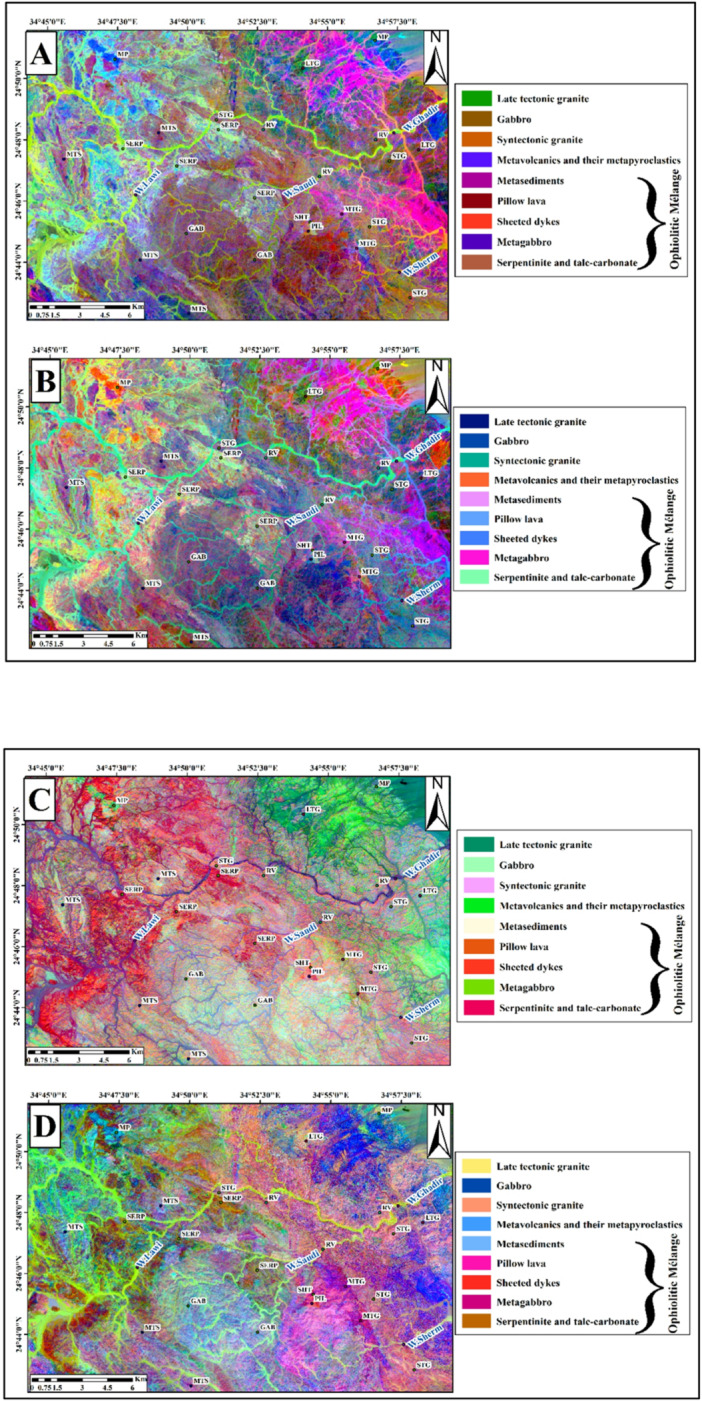
(**A**) Principal Component (PC) composite image (PC5, PC2, PC3 in RGB) from OLI PC bands. (**B**) PC composite image (PC3, PC2, PC5 in RGB) from OLI PC bands. (**C**) PC composite image (PC1, PC2, PC3 in RGB) from Sentinel-2 PC bands. (**D**) PC composite image (PC5, PC4, PC2 in RGB) from Sentinel-2 PC bands. Symbols as in Fig. 3. (By ENVI version 5.6. https://www.l3harrisgeospatial.com/Software-Technology/ENVI).

The best visual results of Landsat 9 are (PC5, PC2 & PC3) in RGB and (PC3, PC2& PC5) in RGB (Fig. 8A & B), and the best results of Sentinel-2 are (PC1, PC2& PC3) in RGB and (PC5, PC4& PC2) in RGB (Fig. 8C & D).

**Table 3:** The eigenvector values of PCA for OLI bands and the highest eigenvector values for each band.

Eigenvector	Band 1	Band 2	Band 3	Band 4	Band 5	Band 6	Band 7
Eigenvalue	0.952661	0.037241	0.006198	0.003687	0.000214	0	0
Eigenvalue %	95.27%	3.72%	0.62%	0.37%	0.002%	0%	0%

**Table 4:** The eigenvector values of PCA for Sentinel-2 bands and the highest eigenvector values for each band.

Eigenvector	B1	B2	B3	B4	B5	B6	B7
Eigenvalue	0.95861374	0.02499185	0.00672971	0.00293499	0.00254958	0.00254958	0.00053363
Eigenvalue%	95.8613738	2.49918473	0.67297145	0.29349856	0.25495835	0.25495835	0.05336337

Eigenvector	B8	B9	B10	B11	B12	B13
Eigenvalue	0.0003854	0.00023717	0.00020752	0.00017788	0.008893	0
Eigenvalue%	0.03854022	0.02371706	0.02075242	0.01778779	0.88939	0

**Fig. 9:**
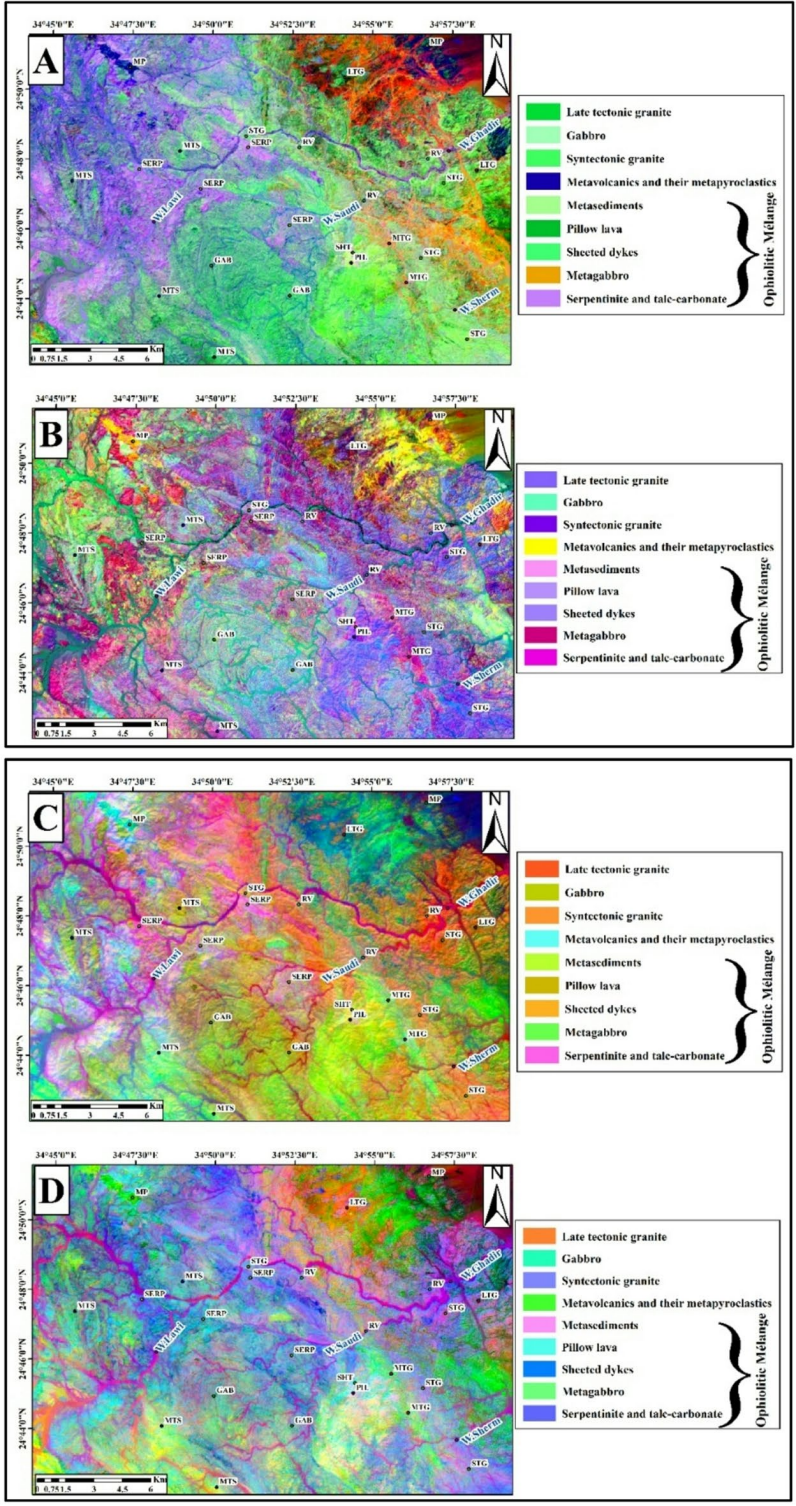
(**A**) MNF composite (MNF5, MNF2, MNF1) of Landsat-9 OLI bands. (**B**) MNF composite (MNF3, MNF4, MNF2) of Landsat-9 OLI bands. (**C**) MNF composite (MNF1, MNF2, MNF3) of Sentinel-2 bands. (**D**) MNF composite (MNF4, MNF2, MNF1) of Sentinel-2 bands. Symbols as in Fig. 3. (By ENVI version 5.6. https://www.l3harrisgeospatial.com/Software-Technology/ENVI).

After examining the Minimum Noise Fraction (MNF) components of Landsat-9 OLI and Sentinel-2, several lithological units were effectively discriminated against. In the Landsat-9 composite of MNF5, MNF2, and MNF1 in RGB (Fig. 9A), metavolcanics and their metapyroclastics appear in dark blue, serpentinite and talc-carbonate rocks in violet, metagabbro in pink, metasediments in green, metavolcanics and their metapyroclastics in yellow, and late-tectonic granite in pale blue when using MNF3, MNF4, and MNF2 in RGB (Fig. 9B). Similarly, the Sentinel-2 MNF composite of MNF1, MNF2, and MNF3 (Fig. 9C) clearly distinguishes serpentinite and talc-carbonate rocks in purple, metagabbro in green, and syn-tectonic granite in orange. In another Sentinel-2 composite (MNF4, MNF2, and MNF1 in RGB; (Fig. 9D), serpentinite and talc-carbonate appear in blue, whereas late-tectonic granite is displayed in orange.

**Fig. 10:**
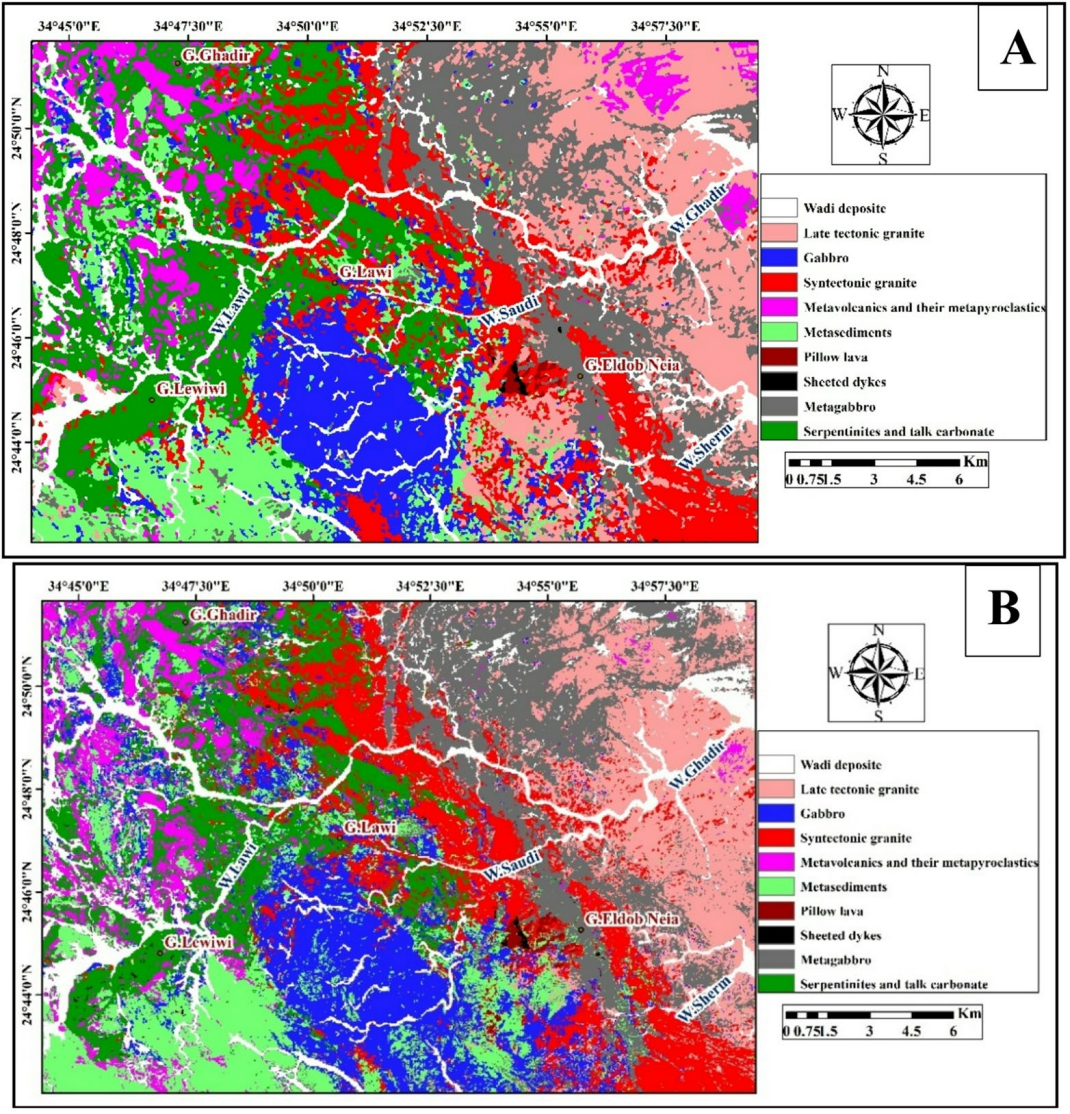
Maximum Likelihood classification of the lithological units in the study area using (**A**) Landsat-9, and (**B**) Sentinel-2 data. (By ENVI version 5.6. https://www.l3harrisgeospatial.com/Software-Technology/ENVI).

The results of the Maximum Likelihood Classification (MLC) applied to OLI imagery and Sentinel-2 data are presented in (Fig. 10A and B), illustrating the rock unit’s discrimination are based on selected Regions of Interest (ROIs). The classification from Landsat-9 (Fig. 10A) successfully identified the main rocks arranged from the oldest to the youngest as follows: serpentinites and talc carbonate (green), metagabbro (gray), sheeted dykes (black), pillow lava (brown), metavolcanics and their metapyroclastics (magenta), metasediments (bale green), syntectonic granite (red), gabbro ((blue) late tectonic granite (light pink), and wadi deposits (white). In contrast, the Sentinel-2 classification (Fig. 10B) reveals a similar lithological distribution but with clearer boundaries and improved differentiation of closely related rock types due to its higher spatial and spectral resolution. Sentinel-2 imagery demonstrates superior capability in delineating detailed geological features, whereas Landsat-9 imagery is advantageous for broader regional mapping. The integration of both datasets enhances the reliability and accuracy of lithological mapping in the G. Lawi area.

The study area was Maximum Likelihood Classification (MLC) which was supervised and checked with 405 independent ground-truth points. Tables 5 & 6 display accuracy measurements of Landsat − 9 and Sentinel − 2 respectively. The Landsat 9 classifier produced an Overall Accuracy (OA) of 90.6% and a Kappa coefficient of 0.89 whilst the Sentinel-2 was better with an OA of 95.8% and a Kappa coefficient of 0.95, which is its better spatial and spectral resolution. The user and producer accuracies of individual lithological units were between 83.3 and 93.8% with Landsat and 90.9 and 98.2% with Sentinel-2 respectively. It is worth noting that Sentinel-2 data improved the discrimination of smaller or more heterogenic units like sheeted dykes, pillow lavas and metavolcanics. These findings are in line with the visual perception of FCC, PCA, and band-ratio, wherein the Sentinel-2-based RGB composites and Landsat-9 were effective in identifying the major ophiolitic units (serpentinite, metagabbro, pillow lavas, metavolcanics, and granitic rocks).

All in all, the comparison reveals that though Landsat9 is an effective scale of the overall mapping task, Sentinel2 is more efficient at the task of detailed lithological discrimination, especially when dealing with small or spectrally sensitive units. These results highlight the benefits of multispectral imagery of high resolution in remote-sensing experiences of geological mapping.

**Table 5:** Accuracy assessment of Landsat-9 OLI MLC showing ground truth samples, correctly classified pixels, and user/producer accuracies.

Class	Ground truth	Correctly classified	User accuracy (%)	Producer accuracy (%)
Serpentinite and talc-carbonate	55	51	92.7	92.7
Metagabbro	60	54	90.0	90.0
Sheeted dykes	15	13	86.7	86.7
Pillow lava	12	10	83.3	83.3
Metasediments	52	46	88.5	88.5
Metavolcanics & metapyroclastics	33	28	84.8	84.8
Syntectonic granite	57	52	91.2	91.2
Gabbro	56	52	92.9	92.9
Late tectonic granite	65	61	93.8	93.8
Overall accuracy	–	–	**90.6%**	–
Kappa coefficient	–	–	**0.89**	–

**Table 6:** Accuracy assessment of Sentinel-2 MSI MLC showing ground truth samples, correctly classified pixels, and user/producer accuracies.

Class	Ground truth	Correctly classified	User accuracy (%)	Producer accuracy (%)
Serpentinite and talc-carbonate	55	54	98.2	98.2
Metagabbro	60	57	95.0	95.0
Sheeted dykes	15	14	93.3	93.3
Pillow lava	12	11	91.7	91.7
Metasediments	52	49	94.2	94.2
Metavolcanics & metapyroclastics	33	30	90.9	90.9
Syntectonic granite	57	55	96.5	96.5
Gabbro	56	55	98.2	98.2
Late tectonic granite	65	63	96.9	96.9
Overall accuracy	–	–	**95.8%**	–
Kappa coefficient	–	–	**0.95**	–

## Field validation, sampling and petrographic studies

**Fig. 11:**
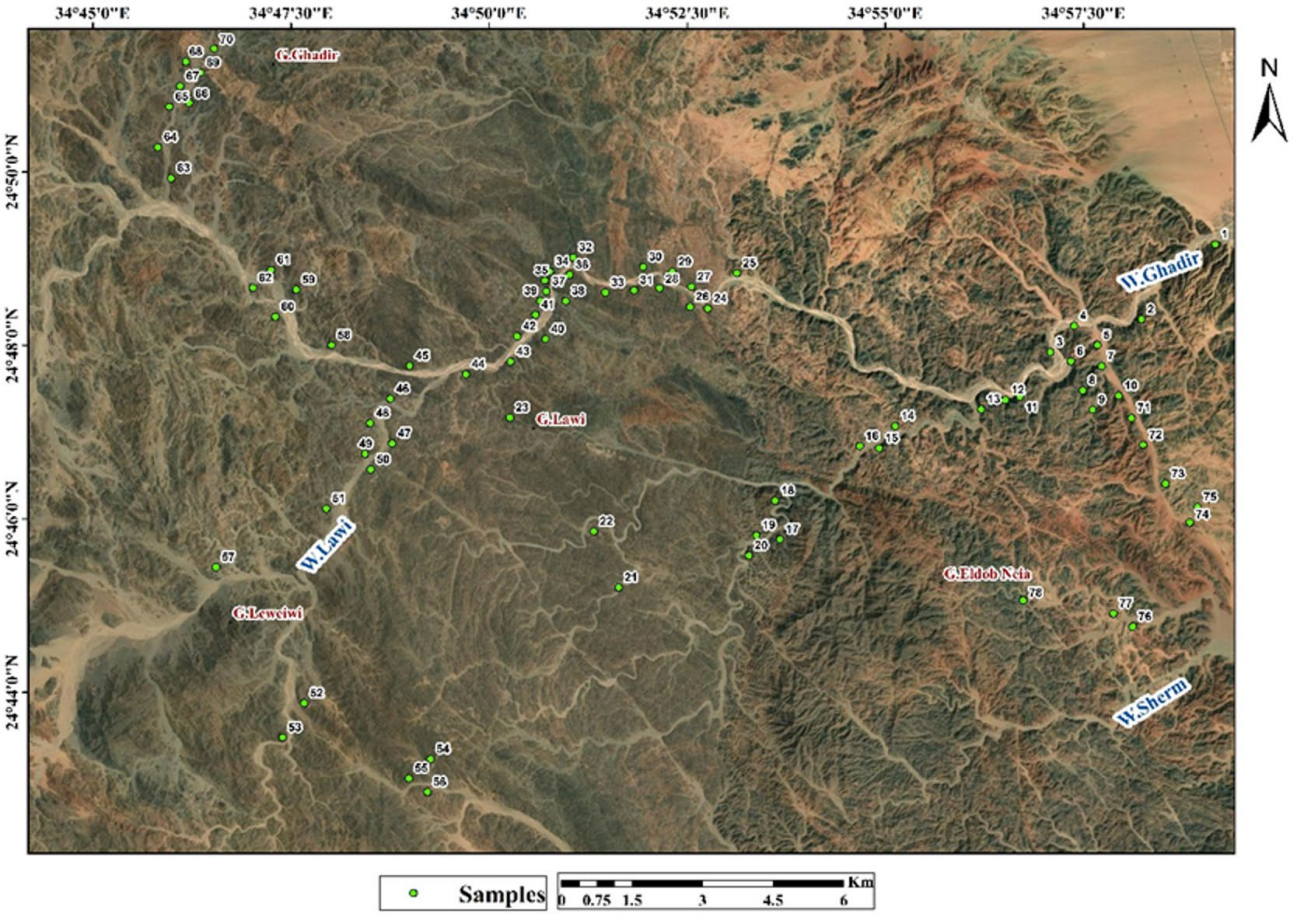
The distribution and locations of the samples collected from the study area. (By ArcGIS v.10.5. https://www.esri.com/en-us/arcgis/products/arcgis-desktop/overview).

A field visit to the study area was conducted to confirm the veracity of the satellite image processing from Landsat and Sentinel. 78 Samples were collected for microscopic cross-sectional examinations (Fig. 11). The Ghadir ophiolitic sequence, located northwest of Gabel El Dob Neia, comprises from base to top, ultramafic rocks (including serpentinite and talc-carbonate), metagabbros, sheeted dykes, pillow lavas, and overlying metasedimentary units^12^.

Field observations indicate that the rock units in the study area, in chronological order from oldest to youngest, comprise serpentinite and talc-carbonate rocks, metagabbro, sheeted dykes, pillow lavas, metasedimentary rocks, metavolcanics with associated metapyroclastics, syntectonic granite, gabbro, late-tectonic granite, and, ultimately, wadi deposits.

### Serpentinite and talc-carbonate

**Fig. 12:**
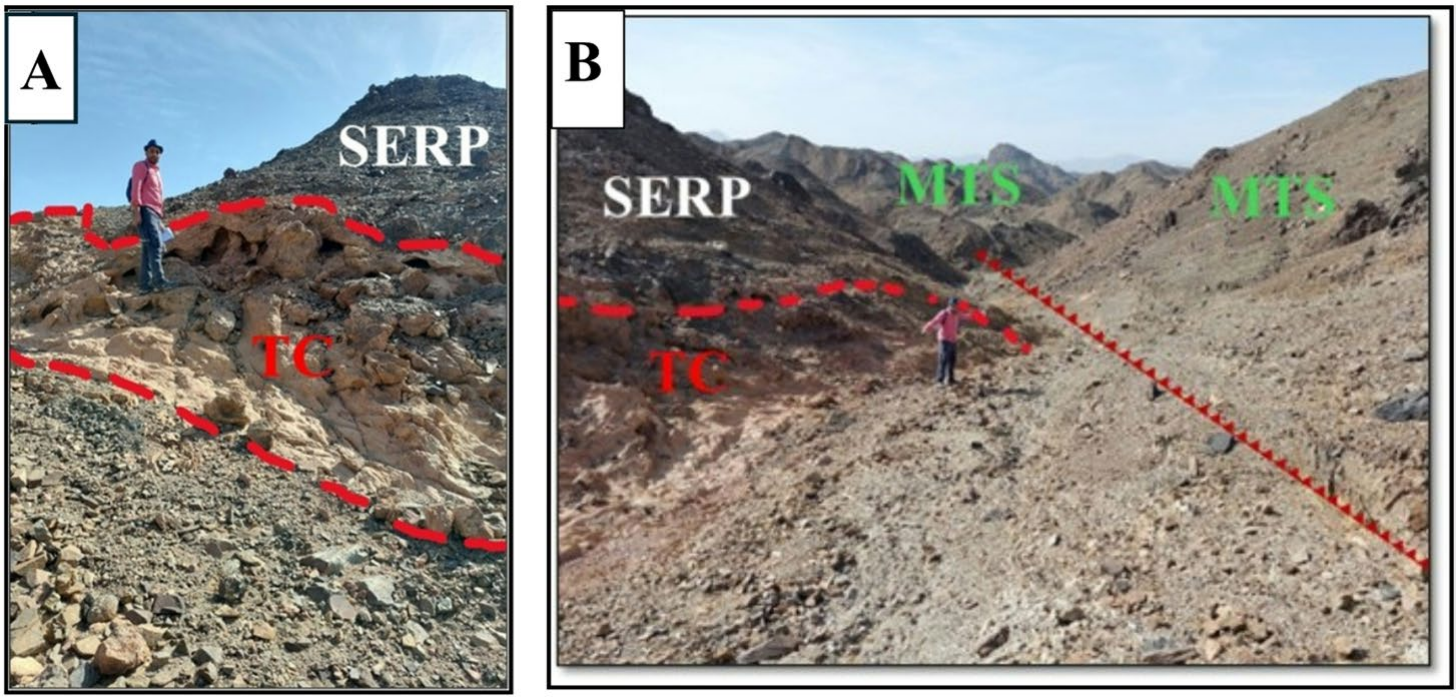
(**A**) Photograph showing the contact between serpentinites (SERP) and talc-carbonates (TC). (**B**) Photograph showing serpentinites (SERP) and talc-carbonates (TC) thrusted by metasediments (MTS).

Serpentinites constitute the lowermost rock unit within the ophiolitic succession exposed at Wadi Ghadir. They occur as relict patches, greenish to brownish gray in color, enclosed within a dense talc-carbonate matrix of buff hue. These serpentinites are overlain by ophiolitic metagabbros, which are mostly coarse-grained and layered at the base, gradually grading upwards into fine-grained hypabyssal gabbros^12^. Serpentinites in Wadi Ghadir ophiolite are thought to be derived from ultramafic source rocks that initially developed along a mid-ocean ridge setting. Subsequently, these rocks were emplaced in a tectonic environment associated with subduction zone processes. Alternatively, they may correspond moderately to highly depleted forearc harzburgite, formed within a supra-subduction zone tectonic setting^71^. Moreover, the ophiolitic assemblage in the Ghadir area is interpreted to have originated in an oceanic back arc basin. Several researchers have proposed a back arc basin origin for the Wadi Ghadir ophiolite^2,10^. In this study area, serpentinite rocks are found throughout the Wadi Ghadir region, in Wadi Lawi forming Gabel Lawi, Wadi Lewiwi forming Gabel Lewiwi and Gabel Ghadir, they are mostly associated with talc-carbonates. Occasionally, they are also found in mélange thrusted by metasediments (Fig. 12A & B).

**Fig. 13:**
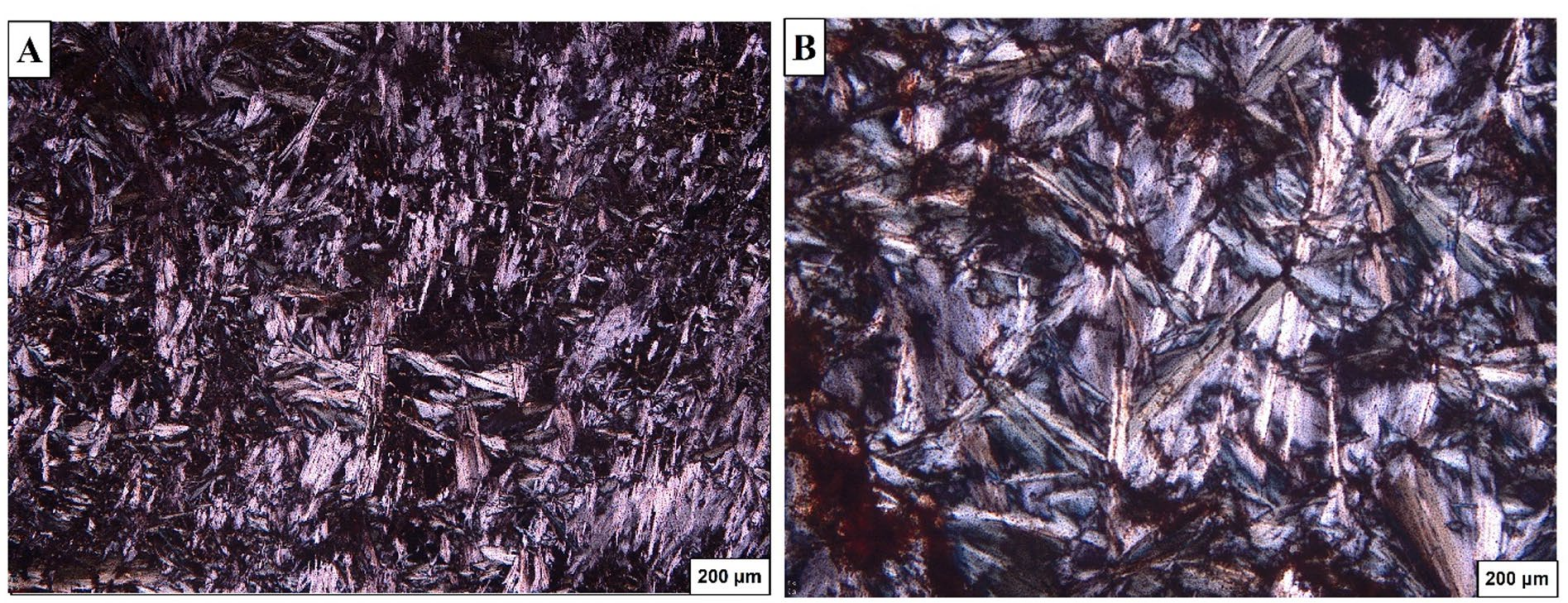
Photomicrographs showing (**A**) Randomly oriented crystals of antigorite in serpentinite. (**B**) Mesh texture of antigorite in serpentinite.

The thin section study indicates that the serpentinites are composed mainly of randomly distributed antigorite which exhibits mech texture (Fig. 13A & B).

### Metagabbro

**Fig. 14:**
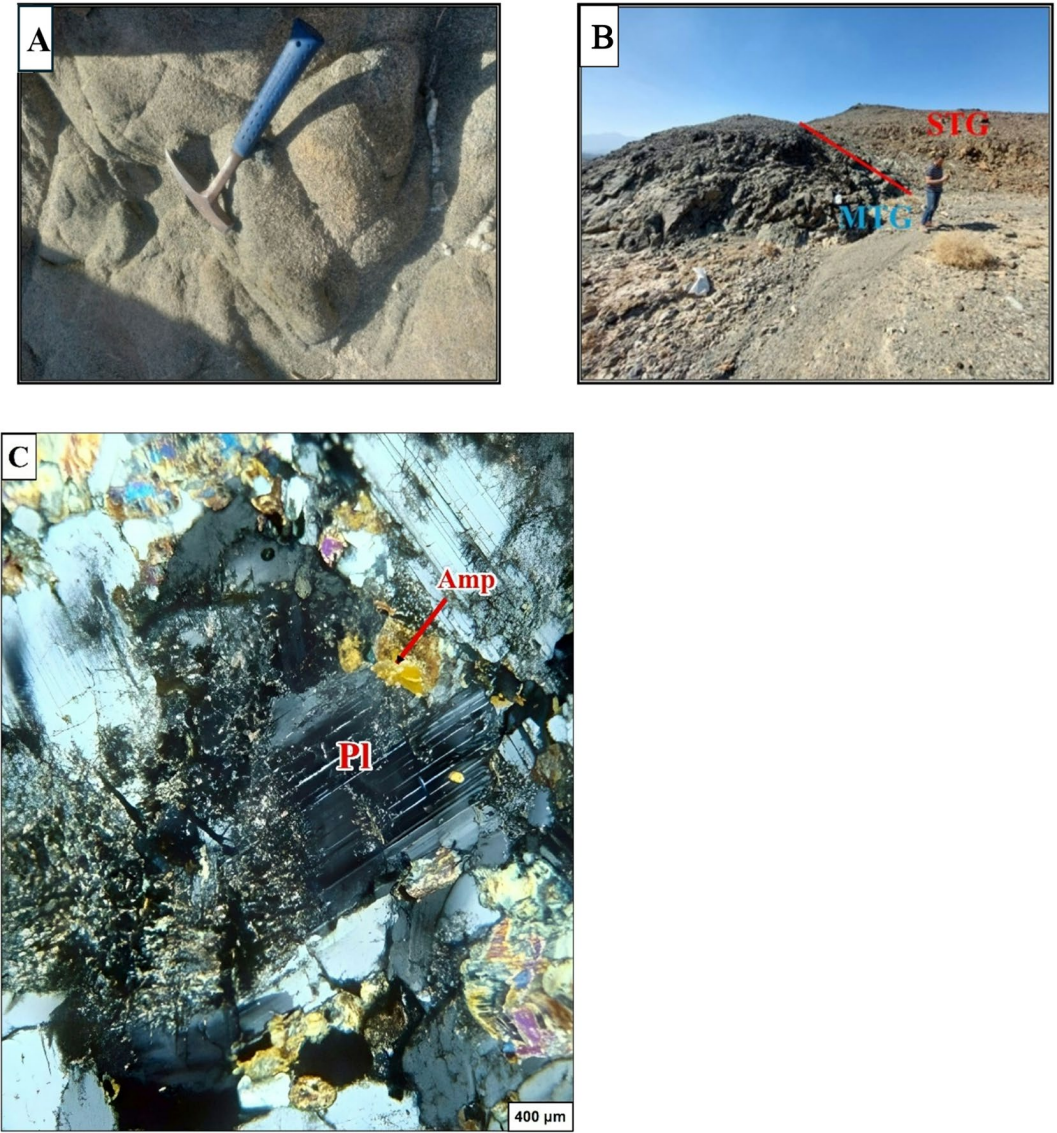
(**A**) Photograph showing coarse-grained in dark greenish to black color of metagabbro in Gabel El Dob Neia. (**B**) Photograph showing the sharp contact between metagabbro (MTG) and syn-tectonic granite (STG) in Gabel El Dob Neia. (**C**) Photomicrograph showing highly altered and saussuritized plagioclase (Pl) in metagabbro, associated with amphibole (Amp).

The largest metagabbroic body found at Gabel El Dob Neia in the southeastern region of this area (Fig. 14A &B). Metagabbro is faulted along Wadi Saudi overlying by sheeted dykes and pillow lava, metagabbro is described as coarse-grained in dark greenish to black color^12^ divided the gabbro into three main horizons, which are moderately tilted to the SW. Layered gabbro occupies the basal part, followed upwards by coarse-grained gabbro, and then fine-grained hypabyssal gabbro at the top. Some metagabbro is enclosed within mélange and is dispersed across various locations along the East part study area.

**Fig. 15:**
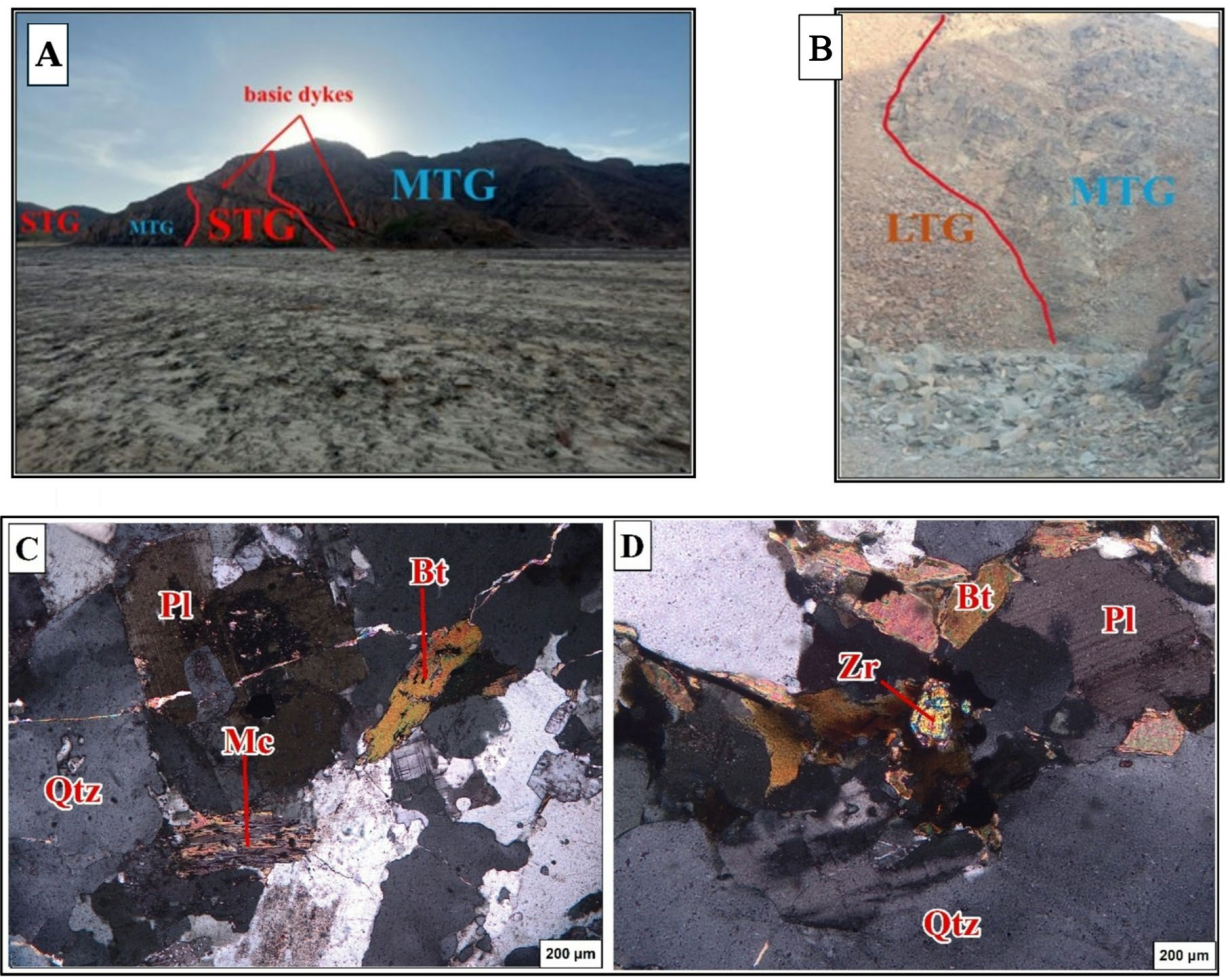
(**A**) Photograph showing syntectonic granite (STG) and basic dykes intruded in Gabel El Dob Neia metagabbro (MTG) and extruded by basic dykes. (**B**) Photograph showing the sharp contact between metagabbro (MTG) and late tectonic granite (LTG) in Gabel El Dob Neia. (**C**) Photomicrograph showing the plagioclase (Pl) and biotite (Bt) associated with quartz (Qtz) and muscovite (Ms), in syntectonic granite. (**D**) Photomicrograph shows euhedral of zircon (Zr) included in fennibiotite (annite) surrounded by pleochroic halos late tectonic granite.

The petrographic examination of the metagabbros shows that composed mainly of plagioclases (Fig. 14C) which is found highly altered and saussuritized, relics of amphibole minerals and epidote are also found. In the northeastern parts of Gabel El Dob Neia metagabbro, there are found intrusions from syn to late tectonic granites marked by sharp intrusive contacts (Fig. 15A, B, C&D).

### Sheeted dykes

**Fig. 16:**
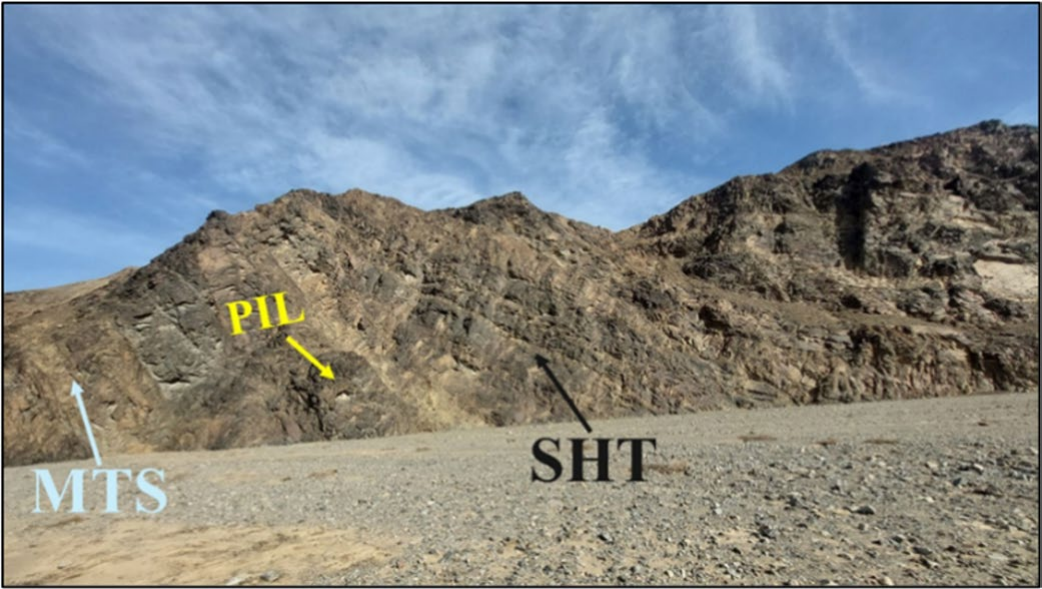
Photograph showing contact between sheeted dykes (SHT), pillow lava (PIL) and metasediments (MTS) (mélange).

The sheeted dykes are prominently renowned and finest observed in the Wadi Ghadir region, where they form a distinct and continuous unit within the ophiolite sequence. This unit is situated between the underlying metagabbro and serpentinite and the overlying pillow lava and metasediments. Sheeted dykes occur as blocks that have been incorporated into the mélange, particularly in Wadi Saudi (Fig. 16).

### Pillow lava

**Fig. 17:**
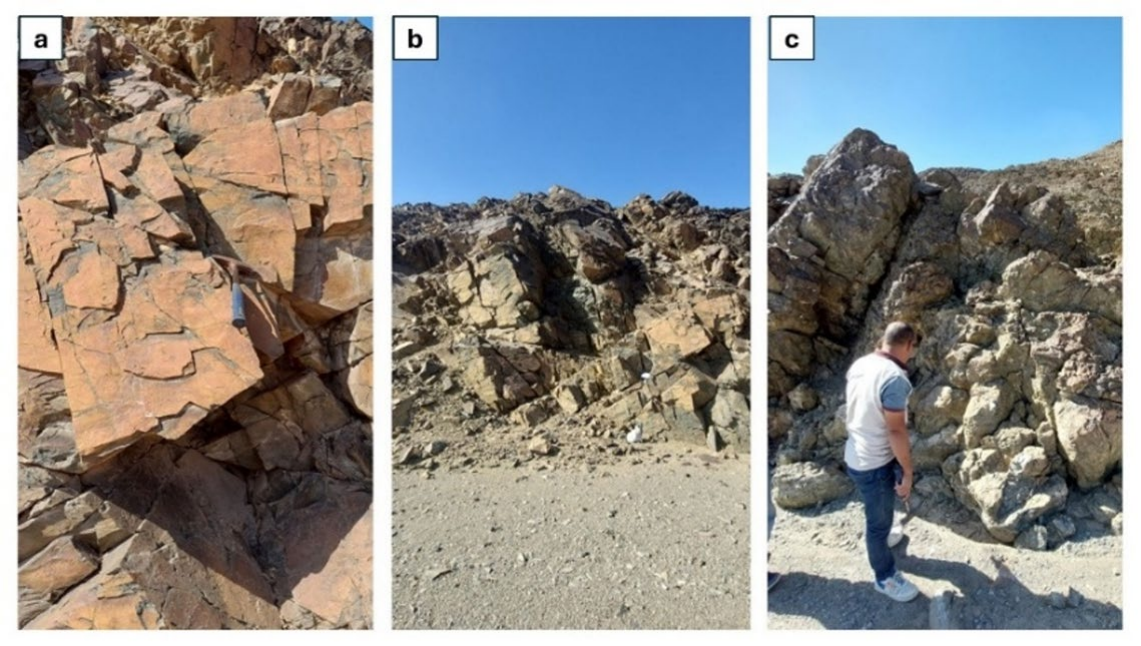
Photograph showing Pillow lava in Wadi Ghadir.

The pillows exhibit a variety of shapes and sizes. Despite significant deformation from jointing and stretching, the pillows retain oval and elliptical forms, with some exhibiting a rounded shape, which directly indicates their underwater eruption. The pillow basalts exhibit greenish to brownish-gray hues and are characterized by a high abundance of vesicles, particularly in the peripheral upper portions, which taper towards the core of the pillow structure. Pillows outcrop in Wadi Saudi of the South East part of the study area following sheeted dykes, in this area the pillows are characterized by brownish color due to hematitic tarnish on the outer surface, the main features of these pillows, which are more deformed and vary in shape from elongate to oval, some are sheared but still retain their original structure (Fig. 17).

### Metasediments

The metasedimentary rocks at Wadi Ghadir are included in the Precambrian basement complex. This complex contains different types of rocks like schists, quartzites, and amphibolites.

**Fig. 18:**
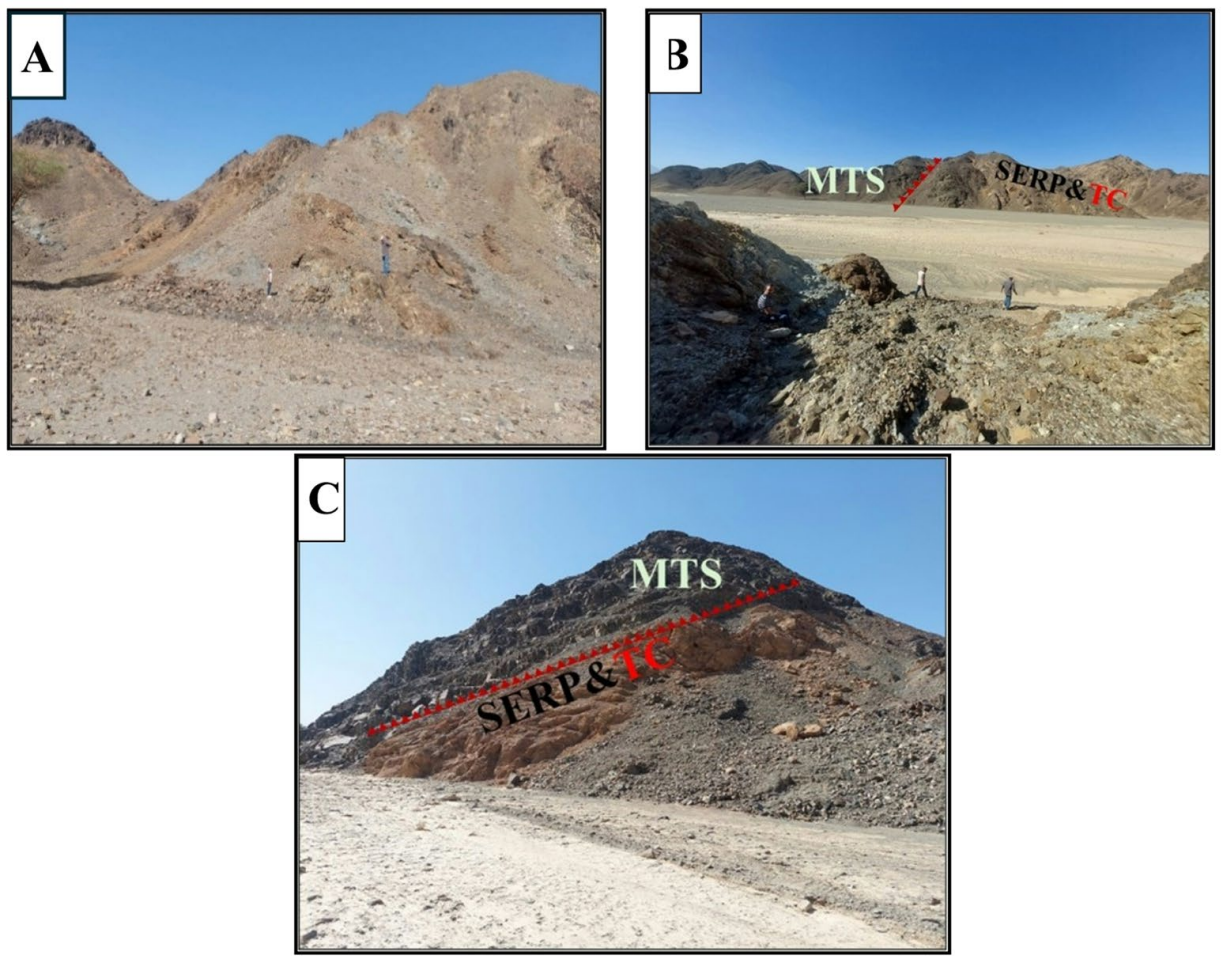
(**A**) Photograph showing metasediments in Wadi Lawi. **(B)** Photograph showing contact between metasediments (MTS) and serpentinite (SERP) and talc-carbonate (TC) in Wadi Ghadir. **(C)** Photograph showing serpentinite (SERP) and talc-carbonate (TC) thrusted by metasediments (MTS) in Wadi Ghadir.

These rocks were first formed as sediments in the sea, probably in the Proterozoic era, before undergoing regional metamorphism. In the study area these rocks are widely distribution in the west, north and central regions of Wadi Lawi and Wadi Ghadir (Fig. 18A and B), in Wadi Ghadir serpentinite and talc-carbonate thrusted by metasediments rock (Fig. 18C).

## Discussion

These studies utilized remotely sensed data collected from Landsat-9 and Sentinel-2 satellites for the recognition of ophiolitic rock units, their tectonic settings, and enhancing the geological mapping of Ghadir region. Various digital image processing methods were applied in this study, such as Maximum Likelihood (ML) supervised classification, along with enhancement and transformation techniques including Minimum Noise Fraction (MNF), Principal Component Analysis (PCA), Band Ratios (BR), and False Color Composites (FCC).

The combination of Bands 7, 5, and 3 in RGB yielded the most suitable False Color Composite (FCC) arrangement for Landsat-9 OLI-2 through the Optimum Index Factor (OIF). The most appropriate combination from Sentinel-2 was found to be Bands 2, 12, and 11 (RGB). These selected combinations readily facilitated the separation of rock units through enhanced visual discrimination of lithological classes.

Lithological discrimination was further improved through the application of Band Ratio (BR) analysis. For landsat-9 data, the optimal RGB band ratio combination (BRC) of (7/5, 5/3, and 3/1) could effectively distinguish between serpentinite and talc-carbonate formations, which manifested as bluish-green units (Fig. 6B). BRC (7/6, 6/4, and 5/4) also differentiate between serpentinite and talc-carbonate, shows in dark brown. The 12/13, 13/6, and 2/1 ratios strongly emphasized these units in the Sentinel-2 imagery. Figure 6B showed a clear representation of metagabbro with light green colors. The Landsat 9 band ratios of 6/7, 6/5, and 6/3 in RGB yielded the best results for distinguishing sheeted dykes from pillow lavas, as shown in (Fig. 6D.)

Lithological mapping has been greatly improved using (PCA) and (MNF) transformations. The use of PC1, PC2, and PC3 in RGB readily enabled the recognition of serpentinite and talc-carbonate in the Sentinel-2 dataset as dark red (Fig. 7C). The RGB combination of PC3, PC2, and PC5 in Landsat 9 also showed orange colors corresponding to the metavolcanics and pyroclastic rocks (Fig. 8B). The gabbroic exposures were shown as pale blue with Sentinel-2 Principal Components combination of 5, 4, and 2, in RGB respectively (Fig. 8D).

The contrast between granitic varieties and metasedimentary deposits was improved through the application of MNF transformation. In RGB composite, bands MNF4, MNF2, and MNF1 of Sentinel-2 illustrated late-tectonic granite in an orange color and metasediments in a blue color (Fig. 9D). Similarly, metavolcanic and pyroclastic units were highlighted in dark blue by Landsat 9 OLI RGB composite of bands MNF5, MNF2, and MNF1 (Fig. 9A).

Improved results were achieved through identifying and separating the lithological rock units of the study area using supervised classification with the Maximum Likelihood (ML) algorithm, although some ambiguity may remain in areas of spectral similarity.

Based on the analysis of Landsat-9 (OLI) and Sentinel-2 satellite images, with field and petrographic studies of Wadi Ghadir area in Southeast Desert of Egypt. The study area revealed the presence of ophiolitic rocks, mainly serpentinite and talc-carbonate, located in the north and central part of Ghadir area, these serpentinite and talc-carbonate are mainly represented by Gebel Lawi and Gebel Lewiwi. Structurally, the serpentinites and talc-carbonate are thrusted by metasediments. They are overlain by metagabbroic units that are exposed in Gabel El Dob Neia in the eastern part of this area. Metagabbro is intruded by metavolcanics and their metapyroclastics, syntectonic granite and late tectonic granite. Sheeted dykes, pillow lava are observed mainly at Wadi Saudi. Metasediments are extensively distributed within the study area in the western, central and the northern parts; they are intruded by fresh gabbro and metavolcanics and their associated metapyroclastics.

**Fig. 19:**
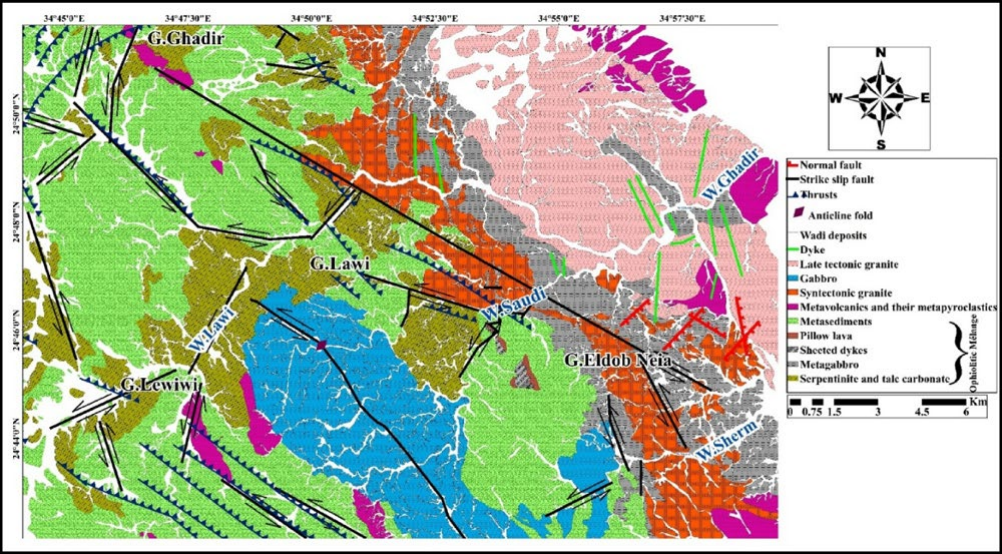
Geologic map of Ghadir area modified after^12,72^ and improved at a scale of 1: 25,000. (By ArcGIS v.10.5. https://www.esri.com/en-us/arcgis/products/arcgis-desktop/overview).

Satellite images interpretation enhanced the mapping accuracy of geological features. As a result, the geological map of Ghadir area was updated after^12,72^ and improved at a scale of 1: 25,000 (Fig. 19).

All in all, the geological knowledge of the Ghadir ophiolitic complex was improved, and the lithological units were clearly defined by the combination of multispectral remote sensing data, advanced digital image processing methods, and field verification studies.

Despite the promising results, several limitations should be acknowledged. The spectral similarity between certain lithological units may have influenced classification accuracy. Additionally, atmospheric interference, terrain shadows, and the limited availability of field validation data in remote zones present challenges to interpretation. These limitations may be addressed in future studies by integrating hyperspectral imagery, more extensive ground-truthing, and geochemical analyses.

## Conclusions

In the research, high-order remote sensing techniques were used, including Minimum Noise Fraction, Principal Component, Analysis band ratioing, and maximum likelihood classification, to narrow down the geological mapping of the Wadi Ghadir ophiolite in the southeastern Egyptian Desert. The data consisted of Landsat − 9 OLI and Sentinel − 2 imagery, and an integrative use of the techniques mentioned above, made it possible to efficiently distinguish between important lithological units, such as serpentinite, talc -carbonate assemblages, metagabbros, sheeted dyke, and pillow lavas. Later fieldwork and petrographic studies supported the results of the remote sensing, and thus, confirmed the structural and lithological features of the research region.

The ensuing 1:25 000 geological map highlights the effectiveness of remote sensing data and orderly field observation to bring out the pertinent results of lithological and structural interpretation. The developed method of mapping will provide a strong basis to future geological and mineral exploration of the study in similar terrains.

Further involvement of field validation and gaining of higher-resolution imagery, as well as increasing the range of spatial coverage are likely to improve research in the future and allow further methodology improvement and more generalized applicability.

## Data Availability

The datasets used and analysed during the current study are available from the corresponding author on reasonable request.
